# The RNA-binding Proteins FMR1, Rasputin and Caprin Act Together with the UBA Protein Lingerer to Restrict Tissue Growth in *Drosophila melanogaster*

**DOI:** 10.1371/journal.pgen.1003598

**Published:** 2013-07-11

**Authors:** Roland Baumgartner, Hugo Stocker, Ernst Hafen

**Affiliations:** Institute of Molecular Systems Biology, ETH Zürich, Wolfgang-Pauli-Strasse, Zürich, Switzerland; Cancer Research UK, United Kingdom

## Abstract

Appropriate expression of growth-regulatory genes is essential to ensure normal animal development and to prevent diseases like cancer. Gene regulation at the levels of transcription and translational initiation mediated by the Hippo and Insulin signaling pathways and by the TORC1 complex, respectively, has been well documented. Whether translational control mediated by RNA-binding proteins contributes to the regulation of cellular growth is less clear. Here, we identify Lingerer (Lig), an UBA domain-containing protein, as growth suppressor that associates with the RNA-binding proteins Fragile X mental retardation protein 1 (FMR1) and Caprin (Capr) and directly interacts with and regulates the RNA-binding protein Rasputin (Rin) in *Drosophila melanogaster*. *lig* mutant organs overgrow due to increased proliferation, and a reporter for the JAK/STAT signaling pathway is upregulated in a *lig* mutant situation. *rin*, *Capr* or *FMR1* in combination as double mutants, but not the respective single mutants, display *lig* like phenotypes, implicating a redundant function of Rin, Capr and FMR1 in growth control in epithelial tissues. Thus, Lig regulates cell proliferation during development in concert with Rin, Capr and FMR1.

## Author Summary

Animal growth is orchestrated by controlled expression of growth-regulatory factors. This regulation is achieved at different molecular levels like transcription, translation initiation, and translational regulation. Whereas transcriptional control and translation initiation of growth components have been well studied, the role of translational control in this process is less well understood. Here, we describe Lingerer (Lig), an UBA domain-containing protein, as a new growth suppressor that associates with the three RNA-binding proteins Fragile X mental retardation protein 1 (FMR1), Rasputin (Rin) and Caprin (Capr). *Drosophila* FMR1, Rin and Capr orthologs are known translational regulators. In *lig* mutants and in *FMR1*, *Capr* and *rin* in combination as double mutants, organ size is increased due to excess proliferation. These data unveil a growth-regulatory function of Lig, and a redundant function of the RNA-binding proteins FMR1, Capr and Rin. Our findings demonstrate the involvement of mRNA-binding proteins in epithelial growth control and may also contribute to a better molecular understanding of the Fragile X mental retardation syndrome.

## Introduction

Understanding how cells and organs control their growth is a major endeavor in developmental biology. In *Drosophila melanogaster* and in mammalian systems, genetic studies have revealed a tight regulation mainly at two different layers. Whereas the Hippo and the Insulin receptor signal transduction pathways alter the transcription of growth-regulatory genes via the co-transcriptional factor Yorkie and the transcription factor FoxO, respectively, TORC1 controls translational initiation via 4EBP and S6K [1]. However, increasing evidence indicates that RNA-binding proteins like Fragile X mental retardation 1 protein (FMR1), mammalian cytoplasmic activation/proliferation associated protein (Caprin) and mammalian Ras-GTPase activating protein SH3 domain binding protein (G3BP) regulate growth and growth factors at the translational level [2]–[5].

In humans, loss of FMR1, a protein with one RGG RNA-binding and two KH domains, causes the most common form of inherited mental retardation, the Fragile X syndrome (FXS). Analysis of FMR1 function in the model organisms mouse and *Drosophila* implicated FMR1 in cell proliferation, cell differentiation and apoptosis in reproductive organs and neuronal tissue via translational regulation of growth-regulatory proteins. For example, FMR1 knockout mice display increased proliferation of adult progenitor/stem cells in two-month-old mice, probably caused by increased protein levels of CDK4, Cyclin D1, and GSK3β as a result of missing translational regulation [2]. In *Drosophila*, FMR1 maintains germline stem cells in ovaries using the miRNA bantam [6], and brains of *FMR1* mutants display increased neuroblast proliferation rates with altered Cyclin E levels [7]. Recently, it was demonstrated that FMR1 associates with the RNA-binding protein Caprin in mice [8] and flies [9] to cooperate in binding to the same mRNA targets (at least in flies [9]).

In humans, Caprin-1 and Caprin-2 comprise the homologous region-1 (HR1) and the homologous region-2 (HR2), which contain RGG motifs. Caprin levels have been correlated with proliferation, e.g. in human T- or B-lymphocytes [10] and the chicken lymphocyte line DT40 [11]. In contrast, inhibition of cell proliferation has been observed e.g. by overexpression of GFP-Caprin-1 in NIH-3T3 cells [10]. Caprin interacts with another RNA-binding protein, G3BP, and binds to growth-associated mRNAs, such as *c-myc* and *cyclin D2* [4]. *Drosophila* Caprin (Capr), which shares the HR1 domain and three RGG motifs but lacks the HR2 domain, cooperates with FMR1 to regulate the cell cycle via the repression of the *CycB* and *Frühstart* mRNAs at the mid-blastula transition in embryos [9].

G3BP consists of an NTF2-like domain and RNA-binding domains (RRM and RGG). It has been implicated in translational control and mRNA decay of growth factors in mammalian model systems. For example, in quiescent Chinese hamster fibroblasts, human G3BP has been reported to bind to the *c-myc* 3′ UTR and to mediate *myc* mRNA decay [12], [13]. Furthermore, in a FilaminC-RasGAP-dependent manner, G3BP regulates two RNA polymerase II kinases, Cdk7 and Cdk9, at the mRNA level to control growth of cardiac myocytes [3]. However, in *Drosophila*, it is not known whether FMR1, Capr and Rasputin (Rin), the fly ortholog of G3BP, regulate cellular growth in epithelial tissues.

In this study, we identify the UBA domain-containing protein Lingerer (Lig) as a novel interaction partner of FMR1, Rin and Capr in flies and present genetic, biochemical and cell biological evidence that a complex of Lig with RNA-binding proteins restricts proliferation in growing tissues. Furthermore, we demonstrate that JAK/STAT signaling is activated in *lig* mutant cells.

## Results

### Lig suppresses tissue overgrowth by regulating cell number in a diet-dependent manner

**Figure 1:**
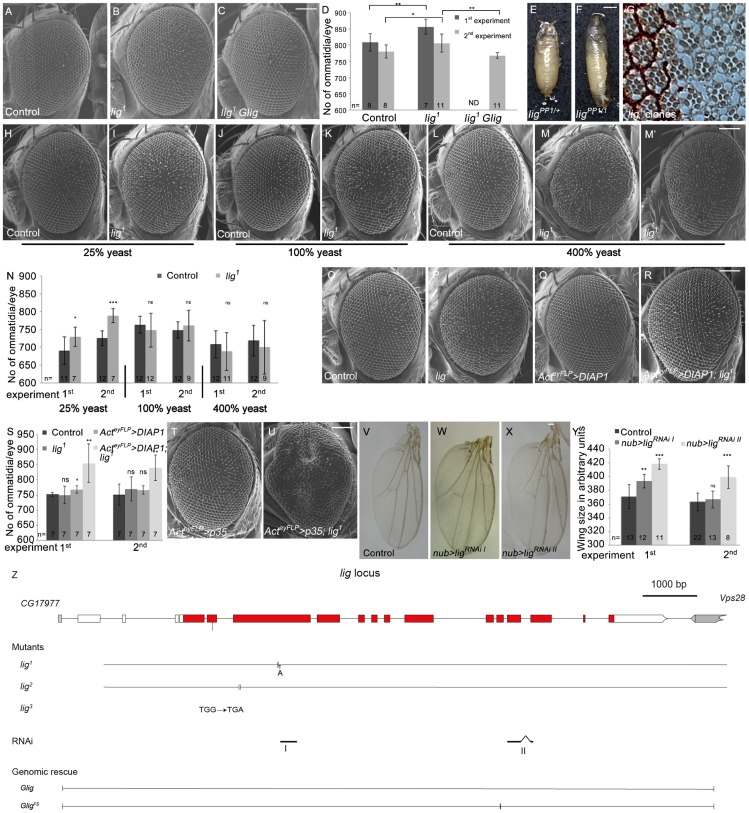
Lig regulates organ size during development. (A–C) Scanning electron micrographs of the control (A), *lig* mutant overgrown eyes (B), and *lig* mutant eyes rescued by one copy of *Glig* (C). The mutant eyes were generated by eyFLP/FRT-mediated mitotic recombination. Scale bar represents 100 µm. (D) Quantification of ommatidia number from two independent experiments. Statistical analyses were done with a Student's t test (two-tailed, unpaired). Error bars indicate the standard deviations, (n) number of organs analyzed. Mean ± s.d. and p-values: Control (808±27 and 780±20), *lig* mutant eyes (856±23; p = 0.0027 and 806±28; p = 0.031) and *lig* mutant eyes with one copy of a genomic rescue transgene for *lig* (ND (not determined) and 768±9; p = 0.0011). (E–F) *lig^1^* in combination with *lig^PP1^*, a *lig* null mutant allele, causes long, slender pupae (F) in comparison to the control (E). Scale bar represents 500 µm. (G) Tangential eye sections of adult *lig^1^* mosaic eyes reveal normal differentiation and cell size in *lig^1^* mutant clones. The *lig^1^* mutant cells are marked by the absence of pigmentation. (H–M) Scanning electron micrographs of adult control and *lig^1^* mutant eyes generated by eyFLP/FRT-mediated mitotic recombination from flies grown on 25% (H–I), 100% (J–K) or 400% (L–M') yeast-containing food. Scale bar represents 100 µm. (N) Statistical analyses as described in Figure 1D: control (690±39 and 726±21) and *lig^1^* mutant (729±27; p = 0.022 and 789±20; p = 2.35E-05) eyes at 25% yeast-containing food, control (763±23 and 749±23) and *lig^1^* mutant (747±47; p = 0.33 and 761±43; p = 0.46) eyes at 100% yeast-containing food, and control (708±38 and 719±43) and *lig^1^* mutant (688±53; p = 0.35 and 700±75; p = 0.48) eyes at 400% yeast-containing food. (O–R) Scanning electron micrographs of adult control (O), *lig^1^* mutant (P), *DIAP1* overexpressing (Q) and *lig^1^* mutant *DIAP1* overexpressing eye (R) generated by eyFLP, Actin-Flp out-Gal4/FRT-mediated mitotic recombination. Scale bar represents 100 µm. (S) Statistical analyses as described in Figure 1D: control (752±7 and 751±35), *lig^1^* mutant (750±29; p = 0.85 and 770±40; p = 0.36), *DIAP1* overexpressing (768±12; p = 0.016 and 766±14; p = 0.34) and *lig^1^* mutant *DIAP1* overexpressing (855±64; p = 0.0053 and 840±42; p = 0.0011) eyes. (T–U) Scanning electron micrographs of adult *p35* overexpressing (T) and *lig^1^* mutant *p35* overexpressing eyes (U) generated by eyFLP, Actin-Flp out-Gal4/FRT-mediated mitotic recombination. Scale bar represents 100 µm. (V–X) Wings overexpressing the indicated UAS transgenes under the control of *nub-Gal4*. Scale bar represents 100 µm. (Y) Statistical analyses of wing area (as described in Figure 1D) of wings overexpressing a control *UAS-RNAi* transgene (371±17.7 and 363±12.9), *UAS-lig^RNAi I^* (393±9.9; p = 1.28E-03 and 366.8±12.4; p = 0.41) and *UAS-lig^RNAi II^* (418.5±7.6; p = 9.47E-06 and 399.3±16.6; p = 2.14E-04). (Z) The *lig* locus (drawn to scale) spans 11.5 kbp and consists of 14 protein-coding exons. *lig^1^* and *lig^2^* are small deletions in the third exon resulting in a frameshift and premature stop codon. *lig^3^* contains a premature stop codon in the second exon. The RNAi lines I and II are specific for exon 3 and for exons 11 and 12, respectively. The genomic rescue construct *Glig* includes 12 kbp. *Glig^FS^* lacks a nucleotide in exon 10 leading to a frameshift and a premature stop. Genotypes: (A) *y w eyFLP*/*y w*; *FRT42 cl w^+^*/*FRT42* (B) *y w eyFLP*/*y w*; *FRT42 cl w^+^*/*FRT42 lig^1^* (C) *y w eyFLP*/*y w*; *FRT42 cl w^+^*/*FRT42 lig^1^*; *Glig* [61B3]/+ (E) *y w*; *lig^PP1^*/*FRT42* (F) *y w*; *lig^PP1^*/*FRT42 lig^1^* (G) *y w hsFlp*/*y w*; *FRT42 w^+^*/*FRT42 lig^1^* (H, J and L) *y w eyFLP*/*y w*; *FRT42 P{SUPor-P}VhaAC45^KG02272^* (cl)/*FRT42* (I, K, M and M') *y w eyFLP*/*y w*; *FRT42 P{SUPor-P}VhaAC45^KG02272^* (cl)/*FRT42 lig^1^* (O) *y w eyFLP*, *Act>CD2>Gal4*/*y w*; *FRT42 P{SUPor-P}VhaAC45^KG02272^* (cl)/*FRT42* (P) *y w eyFLP*, *Act>CD2>Gal4*/*y w*; *FRT42 P{SUPor-P}VhaAC45^KG02272^* (cl)/*FRT42 lig^1^* (Q) *y w eyFLP*, *Act>CD2>Gal4*/*y w*; *FRT42 P{SUPor-P}VhaAC45^KG02272^* (cl)/+; *EP-DIAP1*/+ (R) *y w eyFLP*, *Act>CD2>Gal4*/*y w*; *FRT42 P{SUPor-P}VhaAC45^KG02272^* (cl)/*FRT42 lig^1^*; *EP-DIAP1*/+ (T) *y w eyFLP*, *Act>CD2>Gal4*/*y w*; *FRT42 P{SUPor-P}VhaAC45^KG02272^* (cl)/+; UAS-p35/+ (U) *y w eyFLP*, *Act>CD2>Gal4*/*y w*; *FRT42 P{SUPor-P}VhaAC45^KG02272^* (cl)/*FRT42 lig^1^*; *UAS-p35*/+ (V) *y w*; *nub-Gal4*/+; *UAS-CG1315^RNAi^* (control)/+ (W) *y w*; *nub-Gal4*/+; *UAS-lig^RNAi I^* [86Fb]/+ (X) *y w*; *nub-Gal4*/+; *UAS-lig^RNAi II^* [86Fb]/+.

In a tissue-specific genetic screen for suppressors of tissue growth [14], we recovered a complementation group consisting of three EMS-induced recessive lethal alleles based on increased eye and head size (Figure 1B and 1D). By subsequent mapping in combination with complementation tests, rescue experiments and sequencing, we identified *lig* as the gene responsible for the growth phenotype. *lig* encodes an conserved ubiquitin-associated (UBA) domain-containing protein. All three *lig* alleles, when placed over *lig^PP1^*, a recessive lethal null allele [15], or over the deficiency *Df(2R)Exel7094* uncovering the *lig* locus, resulted in lethality in an early pupal stage, forming long and slender pupae (Figure 1F and Figure S1A and S1B) as described for *lig* null mutants [15]. Both the lethality and the clonal overgrowth phenotype were rescued with one copy of a *lig* genomic rescue construct (*Glig*) (data not shown and Figure 1C, 1D and 1Z) but not with a genomic rescue construct containing a frameshift mutation in exon 10 (*Glig^FS^*) (data not shown and Figure 1Z). Sequence analysis of the *lig* protein-coding sequence of the EMS-induced alleles revealed small deletions (*lig^1^*, *lig^2^*) that result in premature stop codons and a point mutation (*lig^3^*), respectively (Figure 1Z). We conclude that all three *lig* alleles represent null alleles.

To determine whether the *lig* overgrowth phenotype is due to increased cell number or enlarged cell size, we analyzed tangential sections of mosaic compound eyes composed of *lig* mutant clones and wild-type sister clones surrounded by heterozygous cells. In *lig* mutant ommatidia, all cell types were normally differentiated and structured and without cell size defects (Figure 1G), suggesting that the overgrowth phenotype is caused by more cells rather than larger cells. Analysis of adult *lig* mutant eyes revealed a variable ommatidia number. In most cases, the ommatidia number was increased as expected (Figure 1B and 1D), but in some cases, the ommatidia number was equal or even lower than the number in control eyes (Figure 1D). The ommatidia size was not altered in the *lig* mutant eyes (Figure S1C). The increased or reduced ommatidia number of *lig* mutant eyes was completely rescued to a control situation by the presence of the *Glig* transgene (Figure 1C and 1D), thus excluding a second-site mutation as the reason for the variability of the phenotype. Cellular growth is tightly linked to environmental factors like nutrient availability. The variability of the *lig* mutant eye phenotype might thus depend on food conditions. Indeed, animals raised on food with reduced yeast content (25% yeast and 40% yeast, respectively) were delayed and displayed eyes with a constant increase in ommatidia number (Figure 1I, 1N, S1E and S1H). In contrast, animals grown under normal food conditions (100% yeast) displayed a high variability (Figure 1K and 1N), and this effect was even more pronounced in flies from larvae that developed on food with increased yeast content (400%) (Figure 1M, 1M' and 1N).

The diet-dependent phenotype of *lig* mutant eyes may be explained by varying amino acid levels or by altered developmental timing. To test the former possibility, larvae were grown on 40% yeast-containing food supplemented with the milk protein Casein to 100% protein content. However, this food condition did not increase the variability in *lig* mutant eyes (Figure S1G and S1H), excluding altered total amino acid levels as the reason for the variable *lig* phenotype. To investigate the latter possibility, we made use of a *Minute* mutation to reduce the developmental speed under normal food conditions and to generate eyes largely mutant for *lig* with the eyFLP/FRT system. In a second experiment, we induced the developing delay by raising the flies at 18°C. Interestingly, the ommatidia number of *lig* mutant eyes was stably increased only in the *Minute* experiment (Figure S1J and S1K) but variable at 18°C (Figure S1L). However, *lig* mutant eyes of flies raised on 25% yeast-containing food at 18°C produced a stable overgrowth phenotype (Figure S1M), excluding a temperature sensitivity of *lig* mutant cells. These results suggest that the diet-dependent phenotype of *lig* mutant eyes is not dependent on amino acid levels or developmental delay but is probably influenced indirectly by an unknown diet-sensitive process.

To investigate whether the variable phenotype is induced by increased apoptosis in *lig* mutant eyes, we overexpressed the *Drosophila* inhibitor of apoptosis (DIAP1) or baculovirus caspase inhibitor p35 in *lig* mutant eyes to block apoptosis. Indeed, *lig* mutant eyes overexpressing *DIAP1* displayed an increased ommatidia number (Figure 1R and 1S) in comparison to the control (Figure 1O and 1S). Flies with *lig* mutant eyes overexpressing *p35* were dying as pharate adult except for a few escapers that displayed massively overgrown eye structures (Figure 1U). These results are consistent with published data that *DIAP1* overexpression leads to reduced apoptosis rates without developmental consequences [16], [17], whereas *p35* overexpression abolishes virtually all apoptosis but causes an aberrant morphology probably due to “undead cells” that activate compensatory proliferation (reviewed in [17]). We conclude that *lig* mutant cells are sensitive to apoptosis.

To test whether Lig acts as a general growth regulator, we generated two independent RNAi lines against *lig* to downregulate *lig* specifically in different developing tissues (Figure 1Z). The functionality of both RNAi lines was established by ubiquitous expression (using *da-Gal4* as driver) resulting in pupal lethality like *lig* mutants (Figure S1O and S1P) and by compartment-specific reduction of Lig protein levels in the developing eye using the *DE-Gal4* driver line (Figure S1R' and S1S'). Expression of *lig* RNAi in developing eyes increased the ommatidia number (Figure S1U, S1V and S1W) without effecting cell size (Figure S1X) under normal food conditions, similar to the *lig* mutant situation. Consistently, Lig reduction in developing wings by means of RNAi induced overgrowth (Figure 1W, 1X and 1Y), identifying Lig as a general growth regulator.

### Cells overexpressing *lig* undergo apoptosis

**Figure 2:**
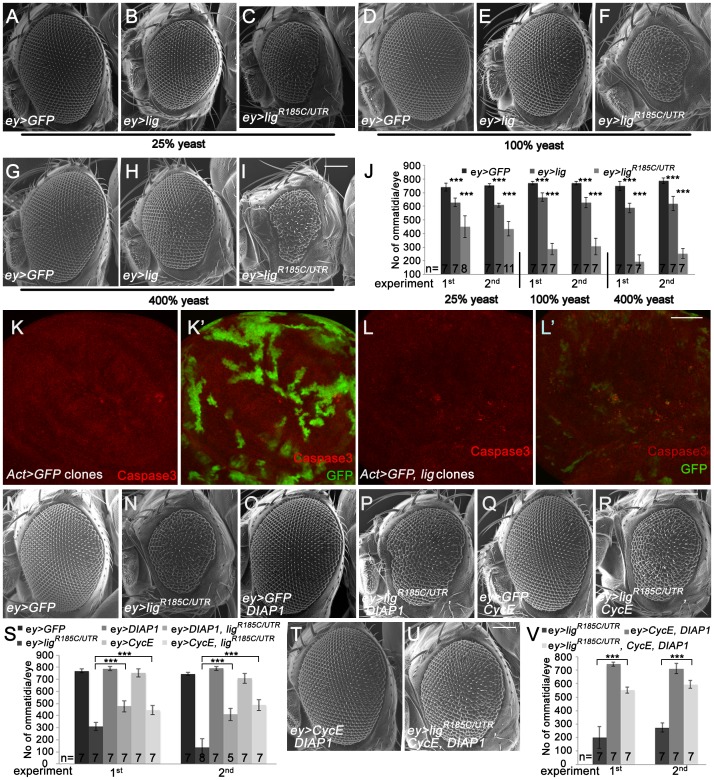
Eyes overexpressing *lig* are reduced in size and are partially rescued by *CycE* and *DIAP1* co-overexpression. (A–I) Scanning electron micrographs of eyes overexpressing the indicated UAS transgenes under the control of *ey-Gal4* during development from flies reared on 25% (A–C), 100% (D–F) and 400% (G–I) yeast-containing food, respectively. Scale bar represents 100 µm. (J) Statistical analyses as described in Figure 1D: *ey>GFP* (741±30 and 753±12), *ey>lig* (628±31; p = 1.66E-05 and 608±14; p = 1.17E-10) and *ey>lig^R185C/UTR^* (448±80; p = 4.35E-06 and 435±54; p = 8.12E-09) eyes at 25% yeast-containing food, *ey>GFP* (768±14 and 768±12), *ey>lig* (666±30; p = 3.03E-05 and 626±39; p = 3.09E-05) and *ey>lig^R185C/UTR^* (287±42; p = 9.50E-09 and 306±62; p = 5.38E-07) eyes at 100% yeast-containing food and *ey>GFP* (748±34 and 785±21), *ey>lig* (588±35; p = 1.55E-06 and 619±53; p = 6.71E-05) and *ey>lig^R185C/UTR^* (193±50; p = 1.18E-10 and 253±36; p = 2.84E-11) eyes at 400% yeast-containing food. (K–L) *lig* overexpressing clones (induced with the Actin-Flp out-Gal4 system and marked by GFP) in wing imaginal discs of third instar larvae undergo apoptosis as judged by Cleaved Caspase-3 staining (red) (L–L') in comparison to the control (K–K'). Scale bar represents 50 µm. (M–R) The reduced size of eyes overexpressing *lig^R185C/UTR^* induced by *ey-Gal4* (N) is partially rescued by co-overexpression of *DIAP1*, an inhibitor of apoptosis (P), or *CycE* (R). Overexpression of *DIAP1* (O) or *CycE* (Q) has no effect on eye size in comparison to the control (M). Scale bar represents 100 µm. (S) Statistical analyses as described in Figure 1D: *ey>GFP* (770±15 and 746±14), *ey>lig^R185C/UTR^* (309±34 and 135±73), *ey>GFP*, *DIAP1* (789±17 and 790±16), *ey>lig^R185C/UTR^*, *DIAP1* (477±47; p = 9.60E-06 and 410±51; p = 7.97E-06), *ey>GFP*, *CycE* (753±33 and 708±42), and *ey>lig^R185C/UTR^*, *CycE* (446±38; p = 1.47E-05 and 488±46; p = 1.01E-07) eyes. (T–U) The rescue of the reduced size of eyes overexpressing *lig^R185C/UTR^* induced by *ey-Gal4* is improved by co-overexpression of *DIAP1* and *CycE* (U) in comparison to the rescue experiment of co-overexpression of *DIAP1* or *CycE*. Co-overexpression of *DIAP1* and *CycE* has no effect on eye size in a wild-type background (T). Scale bar represents 100 µm. (V) Statistical analyses as described in Figure 1D: *ey>lig^R185C/UTR^* (201±83 and 275±35), *ey>CycE*, *DIAP1* (744±14 and 712±37), *ey>lig^R185C/UTR^*, *CycE*, *DIAP1* (551±23; p = 1.45E-05 and 595±29; p = 5.38E-10) Genotypes: (A, D and G) *w*/*y w*; *ey-Gal4*/*UAS-GFP* (B, E and H) *w*/*y w*; *ey-Gal4*/+; *UAS-lig*/+ (C, F, and I) *w*/*y w*; *ey-Gal4*/+; *UAS-lig^R185C/UTR^*/+ (K) *y w hsFLP*/*y w*; *UAS-GFP*/+; *Act>CD2>Gal4*, *UAS-GFP*/+ (L) *y w hsFLP*/*y w*; *Act>CD2>Gal4*, *UAS-GFP*/*UAS-lig* [86Fb] (M–R) first experiment: (M) *w*/*y w*; *ey-Gal4*/*UAS-GFP* (N) *w*/*y w*; *ey-Gal4*/+; *UAS-lig^R185C/UTR^*/+ (O) *w*/*y w*; *ey-Gal4*/*UAS-GFP*; *EP-DIAP1*/+ (P) *w*/*y w*; *ey-Gal4*/+; *UAS-lig^R185C/UTR^*/*EP-DIAP1* (Q) *w*/*y w*; *ey-Gal4*/*UAS-GFP*; *UAS-CycE*/+ (R) *w*/*y w*; *ey-Gal4*/+; *UAS-lig^R185C/UTR^*/*UAS-CycE*; second experiment: (M) *w*/*y w*; *ey-Gal4*/*UAS-GFP; UAS-lacZ* (N) *w*/*y w*; *ey-Gal4*/*UAS-GFP*; *UAS-lig^R185C/UTR^/*+ (O) *w*/*y w*; *ey-Gal4*/*UAS-GFP*; *EP-DIAP1*/+ (P) *w*/*y w*; *ey-Gal4*/+; *UAS-lig^R185C/UTR^*/*EP-DIAP1* (Q) *w*/*y w*; *ey-Gal4*/*UAS-GFP*; *UAS-CycE*/+ (R) *w*/*y w*; *ey-Gal4*/+; *UAS-lig^R185C/UTR^*/*UAS-CycE* (T) *w*/*y w*; *ey-Gal4*/*UAS-CycE; EP-DIAP1*/+ (U) *w*/*y w*; ey-Gal4/*UAS-CycE*; *UAS-lig^R185C/UTR^/EP-DIAP1*.

We next tested the effects of *lig* overexpression in the developing eye under different food conditions using the Gal4/UAS system. To this end, we generated two *UAS-lig* transgenic lines: *UAS-lig* is based on the wild-type coding sequence (based on the release 5.45 of the *Drosophila* genome), and *UAS-lig^R185C/UTR^* encodes a protein version with an amino acid exchange (published as wild type in [15]), including parts of the 5′ and 3′ UTRs of *lig*. Overexpression of the transgenes in the proliferating cells of the developing eye led to smaller adult eyes with fewer ommatidia (Figure 2B, 2C, 2E, 2F, 2H, 2I and 2J), and similar effects were obtained for *UAS-lig^R185C^* (Figure S2B and S2C), suggesting that the amino acid exchange R185C represents a polymorphism. Whereas the overexpression induced by *UAS-lig* mildly reduced the ommatidia number independently of the diet (Figure 2B, 2E, 2H and 2J), *UAS-lig^R185C/UTR^* strongly decreased the eye size in a diet-dependent manner (Figure 2C, 2F, 2I and 2J). The *lig^R185C/UTR^* overexpression eye phenotype was partially rescued in flies grown on 25% yeast-containing food (Figure 2C, compare to 2F and 2I, 2J). Furthermore, *lig* overexpression in the developing wing led to strong reduction of the adult wing size (Figure S2G and S2H).

The ommatidia number of an adult eye depends on the survival and division rate of the cells during eye development. To investigate whether *lig* overexpression results in inappropriate apoptosis of proliferating cells, we analyzed *lig* overexpressing clones in the wing and eye imaginal discs of third instar larvae. Indeed, *lig* overexpressing cells were positive for the apoptosis marker Cleaved Caspase-3 in eye (Figure S2E) and wing imaginal discs (Figure 2L), suggesting that an excess of Lig induces programmed cell death. Note that the effect was stronger in wing imaginal discs in comparison to the eye imaginal disc. Consistently, the reduced eye phenotype induced by *lig^R185C/UTR^* (Figure 2N) was partially rescued by co-overexpression of *DIAP1* (Figure 2P and 2S). In addition, the small eye phenotype was also ameliorated by expression of *CycE* (Figure 2R and 2S). The suppression was further increased by co-overexpression of *DIAP1* and *CycE* (Figure 2U and 2V). These results suggest that the overexpression phenotype of *lig* is caused by increased apoptosis and reduced cell division.

### Lig interacts and co-localizes with the RNA-binding domain-containing proteins FMR1, Rin and Capr

**Figure 3:**
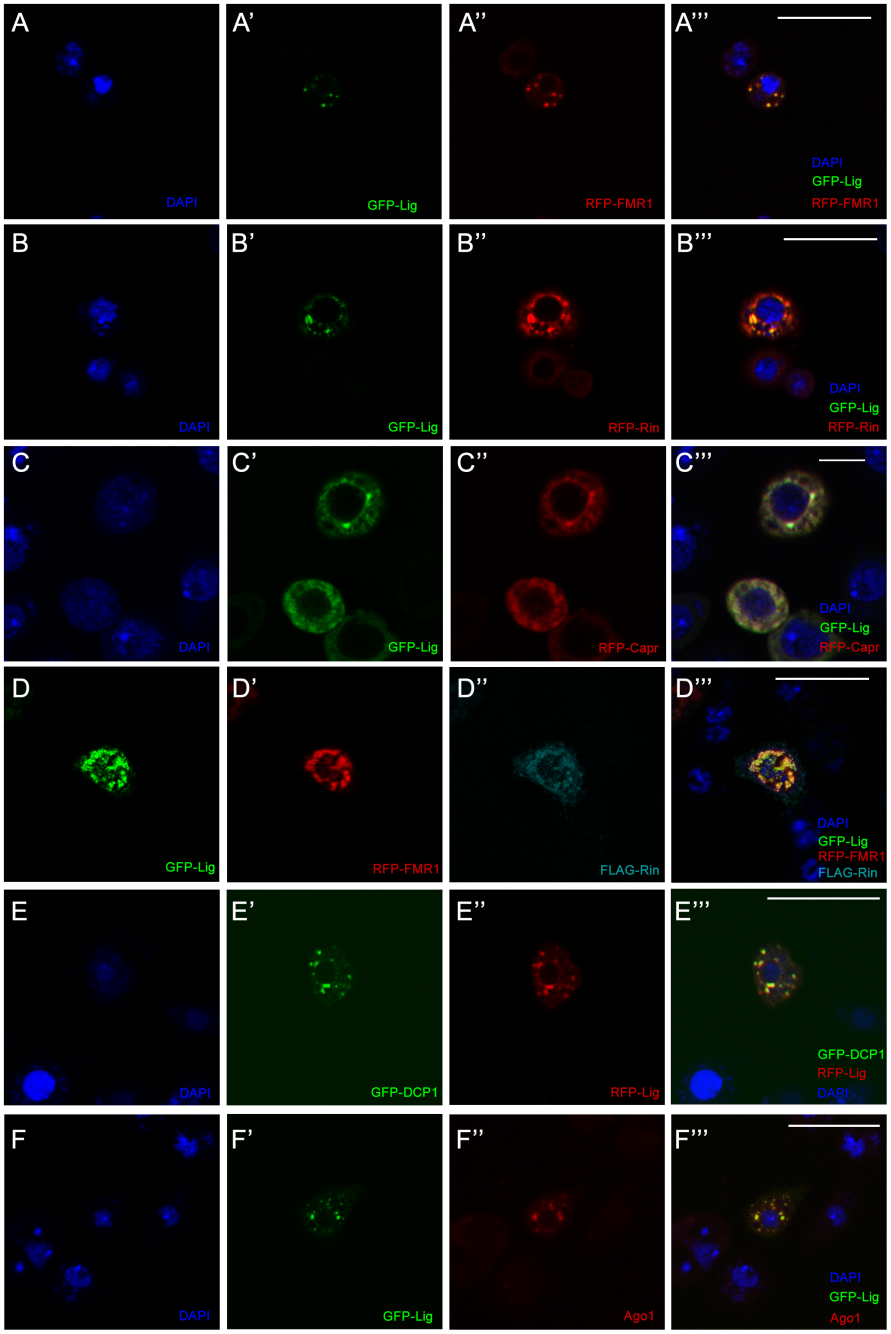
Lig co-localizes with Rin, FMR1, Capr and P-body components in cultured *Drosophila* S2 cells. (A–D''') S2 cells co-transfected with *GFP-lig* (A', A''') and *RFP-FMR1* (A'', A'''), *GFP-lig* (B', B''') and *RFP-rin* (B'', B'''), *GFP-lig* (C', C''') and *RFP-Capr* (C'', C''') and *GFP-lig* (D, D'''), *RFP-FMR1* (D', D''') and *FLAG-rin* (D'', D'''). Cells were stained with DAPI to visualize DNA (blue) and for the FLAG-tag to visualize FLAG-Rin (D'', D'''). Scale bars represent 25 µm. (E–F''') Lig co-localizes with P-body markers in S2 cells transfected with *GFP-DCP1* (E', E''') and *RFP-lig* (E'', E'''), and *GFP-lig* (F', F'''). Cells were stained with DAPI (blue) to visualize DNA and with anti-Ago1 antibody (F'', F'''). Note that endogenous Ago1 is accumulating in GFP-Lig foci. Scale bars represent 25 µm.

To elucidate the function of Lig, we attempted to identify binding partners of Lig using affinity purification coupled with mass-spectrometry (AP-MS). In this experiment, HA epitope-tagged Lig interacted with Rin, FMR1 and DART1, a functional Arginine methyl transferase, in *Drosophila* cultured cells (Table S1). A complex including Lig, Rin, FMR1, Capr, and Orb (oo18 RNA binding), the *Drosophila* cytoplasmic polyadenylation element binding (CPEB) protein, has been identified by co-immunoprecipitation (CoIP) in ovarian extracts using Orb as bait [18]. To confirm the interactions observed in the AP-MS experiment, we performed co-localization experiments with overexpressed epitope-tagged proteins in cultured *Drosophila* cells. Lig, FMR1 and Rin localized in punctae in the cytoplasm and were not observed in the nucleus (Figure S3A, S3B and S3C). Co-overexpression of Lig, FMR1 and Rin (pairwise and all three together) (Figure 3A''', 3B''' and 3D''') or Lig and Capr revealed co-localization in cytoplasmic punctae (3C'''). In contrast, no co-localization was observed between Lig and DART1 (Figure S3F'''). To test whether the endogenous proteins of Lig, Capr, FMR1 and Rin co-localize in cultured *Drosophila* cells, we transfected the cells with a Cherry-tagged Rin genomic rescue transgene (*Grin^Cherry^*) and performed antibody stainings to visualize Lig, FMR1 and Capr. Rin-Cherry was homogeneously distributed in the cytoplasm (Figure S3H). In some cases, we observed discrete punctae in the cytoplasm suitable for co-localization studies. Indeed, Lig, FMR1 and Capr co-localized with these punctae (Figure S3I''', S3J''' and S3K'''). However, when we analyzed Lig and Capr localization in cultured *Drosophila* cells by antibody staining, Lig and Capr co-localized only within bigger dots in few cells (S3L''').

FMR1 interacts with the RISC complex [19] and co-localizes with a P-body marker in cultured *Drosophila* cells [20]. The co-localization of Lig and FMR1 suggested that Lig also localizes to P-bodies. Therefore, we tested whether Lig punctae overlap with the P-body markers DCP1 and Ago1 [21] using co-overexpression and antibody staining, respectively. Indeed, RFP-Lig and GFP-DCP1 co-localized in cultured *Drosophila* cells (Figure 3E'''), and GFP-Lig punctae were positive for Ago1 antibody staining (Figure 3F'''). Note that Ago1 was evenly distributed in small punctae in the cytoplasm of untransfected cells (Figure S3G) but accumulated in GFP-Lig dots of transfected cells. We conclude that Lig localizes to P-bodies in cultured *Drosophila* cells.

**Figure 4:**
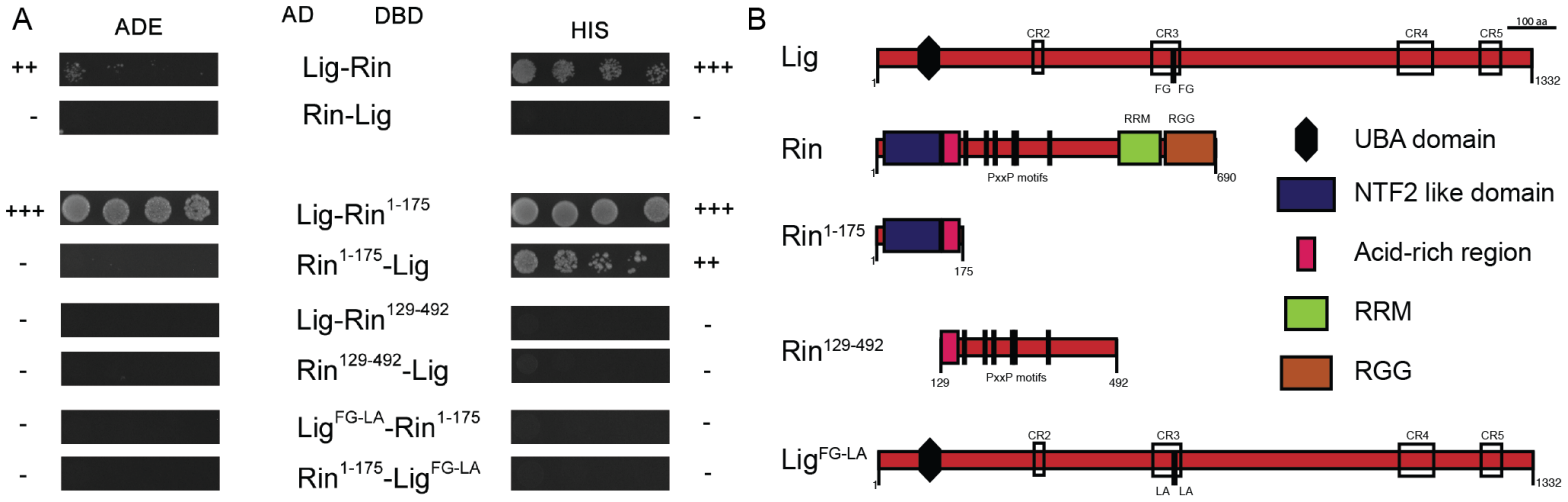
Lig interacts with Rin in Y2H experiments. (A) Y2H interactions between Lig, Lig^FG-LA^, Rin, Rin^1–175^ and Rin^129–492^. Lig fused to the DBD interacts with Rin fused to the AD. Lig fused to the AD does not interact with Rin fused to the DBD. Lig binds to the fragment Rin^1–175^ containing the NTF2-like domain but not to the fragment Rin^129–492^ containing the PxxP motifs. Lig with mutated putative binding sites for Rin (FG repeat mutated to LA repeat; Lig^FG-LA^) does not interact with Rin^1–175^. (B) A linear protein model of Lig with the predicted UBA domain, the conserved regions (CR2-4) and the FG repeat is presented at the top. Linear protein models of Rin with the NTF2-like domain, the acid-rich region, the PxxP motifs and the RNA-binding motifs (RNA recognition motif (RRM) and arginine-glycine rich region (RRG)) as well as the Rin fragments Rin^1–175^ and Rin^129–492^ are presented in the middle. A linear protein model of Lig with mutated FG (Lig^FG-LA^) repeat is presented at the bottom.

Based on the localization experiments, we focused on the interaction between Lig, FMR1, Rin and Capr. To test for direct interactions, we performed a yeast two-hybrid (Y2H) assay. Lig, FMR1, Rin, and Capr were N-terminally fused to the activation domain (AD) or to the DNA-binding domain (DBD) of Gal4, respectively, and tested for autoactivity (Figure S4A). We used plates lacking adenine (ADE) to test for strong interactions and plates lacking histidine (HIS) for weak interactions. Lig interacted with Rin but not with FMR1 or Capr in the Y2H assay (Figure 4A, and data not shown), identifying Rin as a direct interaction partner of Lig. The interaction between Lig and Rin was only visible when Lig and Rin were tagged with the AD and the DBD, respectively. To identify the interaction domain in Rin, we generated three Rin protein fragments: Rin^1–175^ consisting of the NTF2-like domain and the acid-rich region, Rin^129–492^ containing the acid-rich region and six PxxP motifs, and Rin^445–690^ containing the RNA recognition motif (RRM) and Arginine-Glycine rich region (RGG) (Figure 4B and data not shown). In the Y2H assay, the fragment encompassing the NTF2-like domain interacted with Lig (Figure 4A). Proteins with NTF2-like domains like NTF2, TAP15/p15 and Importinβ have been shown to bind to FxFG, FG and GLFG repeats [22]–[24]. Recently, the structure of the Rin NTF2-like domain was resolved but binding sites for the FG motifs are not conserved [25]. However, analysis of Lig, which consists of a predicted UBA domain at the N-terminus and four conserved regions (CR2-4) [15] (Figure 4B), revealed two FGs in close proximity within the CR3 that could serve as a binding site for the NTF2-like domain of Rin. Indeed, when we mutated the FG repeat to a Leucine-Alanine (LA) repeat in Lig, the interaction between Rin and Lig was completely abolished (Figure 4A and 4B). Thus, Rin is a direct interaction partner of Lig, and the interaction occurs via the NTF2-like domain of Rin and the FG repeat of Lig.

### FMR1, Rin and Capr synergize in growth control to inhibit proliferation in epithelial tissues

**Figure 5:**
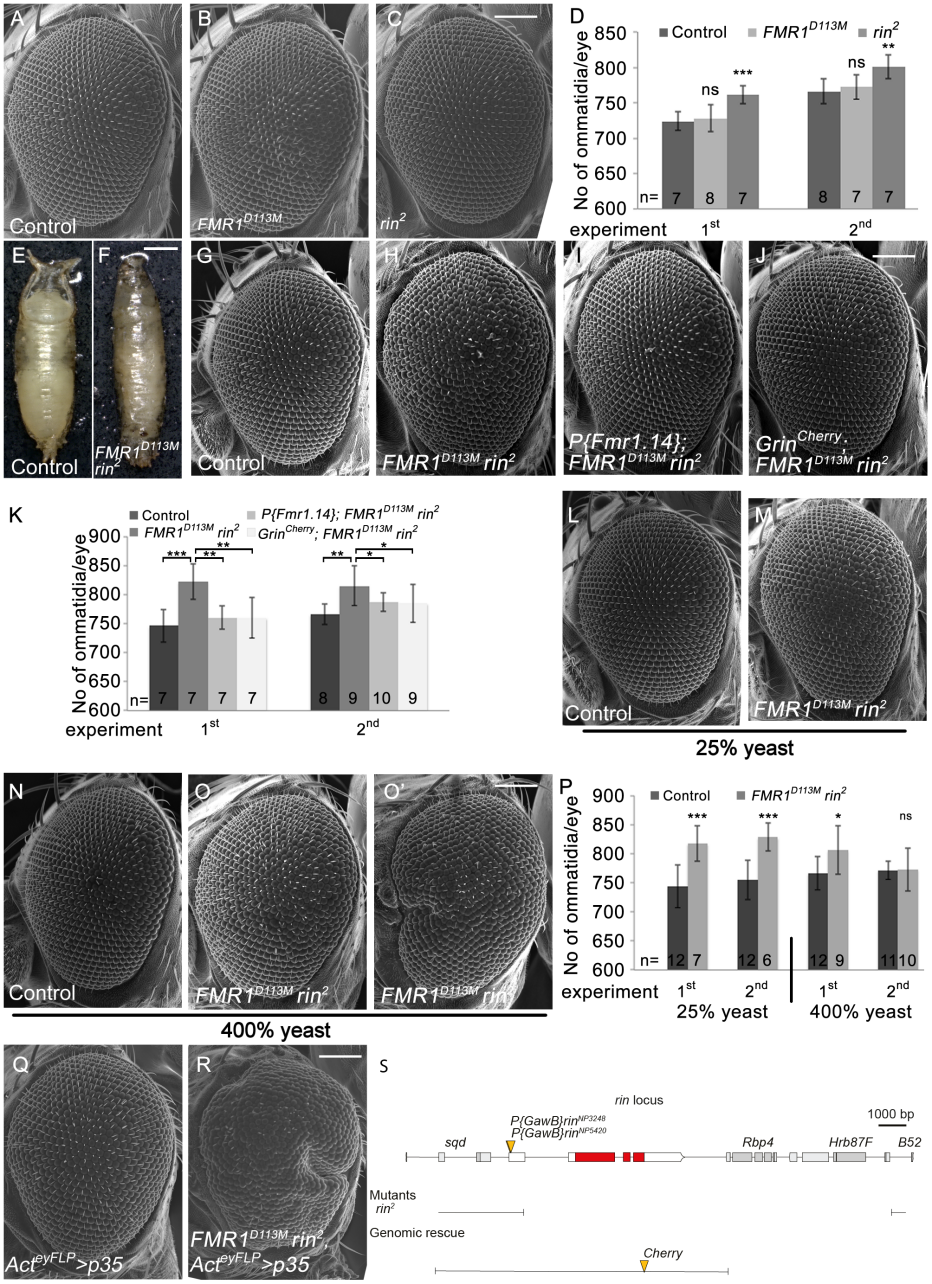
Rin cooperates with FMR1 to suppress growth. (A–C) Scanning electron micrographs of adult control (A), *FMR1^D113M^* (B) and *rin^2^* (C) mutant eyes generated by eyFLP/FRT-mediated mitotic recombination. Scale bar represents 100 µm. (D) Statistical analyses as described in Figure 1D: control (724±13 and 766±18*), *FMR1^D113M^* (728±19; p = 0.68 and 772±17; p = 0.56) and *rin^2^* (761±13; p = 1.85E-04 and 801±17; p = 2.16E-03). (E–F) *FMR1^D113M^ rin^2^* homozygous animals die in the early pupal stage forming long, slender pupae (F) in comparison to the control (E). Scale bar represents 500 µm. (G–J) Scanning electron micrographs of adult eyes from control (G), *FMR1^D113M^ rin^2^* (H), *P{Fmr1.14}*; *FMR1^D113M^ rin^2^* (I) and *Grin^Cherry^*; *FMR1^D113M^ rin^2^* (J) mutant eyes generated by eyFLP/FRT-mediated mitotic recombination. Scale bar represents 100 µm. (K) Statistical analyses as described in Figure 1D: control (746±28 and 766±18*), *FMR1^D113M^ rin^2^* (822±31; p = 4.58E-04 and 815±34; p = 2.79E-03), *P{Fmr1.14}*; *FMR1^D113M^ rin^2^* (760±20; p = 1.17E-03 and 787±16; p = 4.45E-02) and *Grin^Cherry^*; *FMR1^D113M^ rin^2^* (760±35; p = 4.94E-03 and 785±33; p = 7.87E-02). * identical control in the second experiment of (D) and (K). (L–O) Scanning electron micrographs of adult control and *FMR1^D113M^ rin^2^* eyes generated by eyFLP/FRT-mediated mitotic recombination from flies grown on 25% (L–M) or 400% (N–O') yeast-containing food. Scale bar represents 100 µm. (P) Statistical analyses as described in Figure 1D: control (744±37 and 755±34) and *FMR1^D113M^ rin^2^* (817±31; p = 3.35E-04 and 828±24; p = 1.07E-04) mutant eyes at 25% yeast-containing food and the control (766±29 and 771±15) and *FMR1^D113M^ rin^2^* (806±41; p = 0.028 and 773±37; p = 0.88) mutant eyes at 400% yeast-containing food. (Q–R) Scanning electron micrographs of adult *p35* overexpressing (Q) and *FMR1^D113M^ rin^2^* mutant *p35* overexpressing eyes (R) generated by eyFLP, Actin-Flp out-Gal4/FRT-mediated mitotic recombination (Q–R). Scale bar represents 100 µm. (S) The *rin* locus (drawn to scale) spans 8.2 kbp and consists of three protein-coding exons (red filled boxes). The allele *rin^2^* represents a 13 kbp deficiency uncovering *rin*, *Rbp4* and *Hrb87F*. The two P-elements *P{GawB}rin^NP3248^* and *P{GawB}rin^NP5420^* are inserted in the 5′ UTR of *rin*. The genomic rescue construct *Grin^Cherry^* includes 10.3 kbp. Genotypes: (A, G, L and N) *y w eyFLP*/*y w*; *FRT82 cl w^+^*/*FRT82* (B) *y w eyFLP*/*y w*; *FRT82 cl w^+^*/*FRT82 FMR1^D113M^* (C) *y w eyFLP*/*y w*; *FRT82 cl w^+^*/*FRT82 rin^2^* (H, M, O and O') *y w eyFLP*/*y w*; *FRT82 cl w^+^*/*FRT82 FMR1^D113M^ rin^2^* (I) y w *eyFLP*/*y w*; *P{Fmr1.14}* [44E3]/+; *FRT82 cl w^+^*/*FRT82 FMR1^D113M^ rin^2^* (J) *y w eyFLP*/*y w*; *Grin^Cherry^* [44F]/+; *FRT82 cl w^+^*/*FRT82 FMR1^D113M^ rin^2^* (Q) *y w eyFLP*, *Act>CD2>Gal4*/*y w*; *UAS-p35*/+; *FRT82 cl w^+^*/+ (R) *y w eyFLP*, *Act>CD2>Gal4*/*y w*; *UAS-p35*/+; *FRT82 cl w^+^*/*FRT82 FMR1^D113M^ rin^2^*.

The physical interaction of Lig with the RNA-binding domain-containing proteins Rin, FMR1 and Capr suggested that Lig is involved in an RNA-regulatory network and regulates growth via Rin, FMR1 and Capr. To investigate this possibility, we first focused on Rin and FMR1 that we identified as binding partners in the AP-MS experiments. No growth phenotypes in *Drosophila* epithelial tissues have been reported for *rin* and *FMR1* mutants so far. To analyze a putative growth function of FMR1 and Rin, we used the *FMR1* null mutant alleles *FMR1^D113M^* and *FMR1^D50M^* and the *rin* null mutant allele *rin^2^*, respectively. Flies homozygous for the *FMR1* alleles or the *rin^2^* allele are viable and do not display obvious growth phenotypes. Note that *rin^2^* contains a 13 kbp deletion removing the complete coding sequence of *rin* as well as the *Rbp4* and *Hrb87F* loci (Figure 5S). Hence, we attempted to identify additional *rin* alleles to exclude secondary effects of *Rbp4* and *Hrb87F*. We wondered whether the P-elements *P{GawB}rin^NP3248^* and *P{GawB}rin^NP5420^*, inserted in the 5′ UTR of *rin*, are *rin* alleles and tested them with a Cherry-tagged Rin genomic rescue transgene (*Grin^Cherry^*). In the course of the *rin* rescue experiments, we identified a Rin dosage-dependent regulation of *Grin^Cherry^*. Whereas Rin-Cherry was upregulated in *rin* mutant clones, Rin-Cherry was slightly downregulated in the sister clone, suggesting a tight regulation of *rin* to achieve wild-type levels of the gene product (Figure 5S, S5A' and S5A''). Indeed, cells homozygous for either of the P-elements upregulate Rin^Cherry^, verifying both P-elements as *rin* alleles (Figure S5B', S5B'', S5C' and S5C''). Both P-elements placed over *rin^2^* were viable without phenotypic alterations (data not shown). In the eyFLP/FRT experiment, *rin^2^* but not the *rin* P-elements or *FMR1* mutant eyes showed an increase in ommatidia number (Figure 5B–5D, S5E–S5G) under normal food conditions (100% yeast). In contrast to the *lig* mutant phenotype, we never observed a variability of the ommatidia number in *FMR1* or *rin* mutant eyes under normal food conditions. Thus, the single mutant phenotypes of *FMR1* and *rin* did not display growth phenotypes similar to the effects caused by *lig*.

In a next step, we tested for functional redundancy using a double mutant situation of *rin* and *FMR1* since FMR1 and Rin are dispensable for viability and are both RNA-binding proteins that co-localize in cultured *Drosophila* cells. Most *rin^2^*, *FMR1^D113M^* homozygous larvae died at an early stage but few escapers that reached the early pupal stage formed long, slender pupae (Figure 5F), reminiscent of the *lig* null mutant phenotype. Consistently, *P{GawB}rin^NP3248^*, *FMR1^D50M^* or *P{GawB}rin^NP5420^*, *FMR1^D50M^* over *rin^2^*, *FMR1^D113M^* also resulted in long slender pupae (Figure S5K and S5Q). Note that pupae with the P-element *P{GawB}rin^NP3248^* reached a late pupal stage, and pupae with the P-element *P{GawB}rin^NP5420^* developed into adult flies that were dying soon after eclosion. In both combinations, the pupal phenotype and the lethality were rescued by the presence of the *Grin^Cherry^* transgene (Figure S5M, S5S and data not shown). The two P-elements are therefore likely to represent hypomorphic alleles of *rin*.

We then tested for a redundant function of FMR1 and Rin in growth control by using the eyFLP/FRT system to generate *FMR1*, *rin* double mutant eyes under different food conditions. *rin^2^*, *FMR1^D113M^* double mutant eyes consisted of more ommatidia under normal food conditions (Figure 5H and 5K). The double mutant phenotype was rescued to a *rin^2^* and *FMR1^D113M^* like phenotype by the presence of a *FMR1* genomic rescue transgene (*P{Fmr1.14}*) (Figure 5I and 5K) and a genomic *rin* rescue transgene (*Grin*) (Figure S5T), respectively, suggesting a complete rescue for FMR1 and Rin function. However, by the presence of *Grin^Cherry^* the double mutant eyes were not completely rescued to a *FMR1^D113M^* mutant situation (Figure 5J and 5K), suggesting that the C-terminal tag impairs Rin activity. Like in *lig* mutants, *FMR1 rin* double mutant eyes were stabilized at reduced food conditions (25% yeast) (Figure 5M and 5P) but variable at rich food (400% yeast) (Figure 5O, 5O' and 5P). Furthermore, overexpression of *p35* in *FMR1 rin* double mutant eyes resulted in pharate adults except for some escapers displaying massively overgrown eyes (Figure 5R). Taken together, *FMR1 rin* double mutant eyes, but not the single mutants, displayed a *lig* like phenotype, suggesting a functional relationship between *lig*, *FMR1* and *rin*.

**Figure 6:**
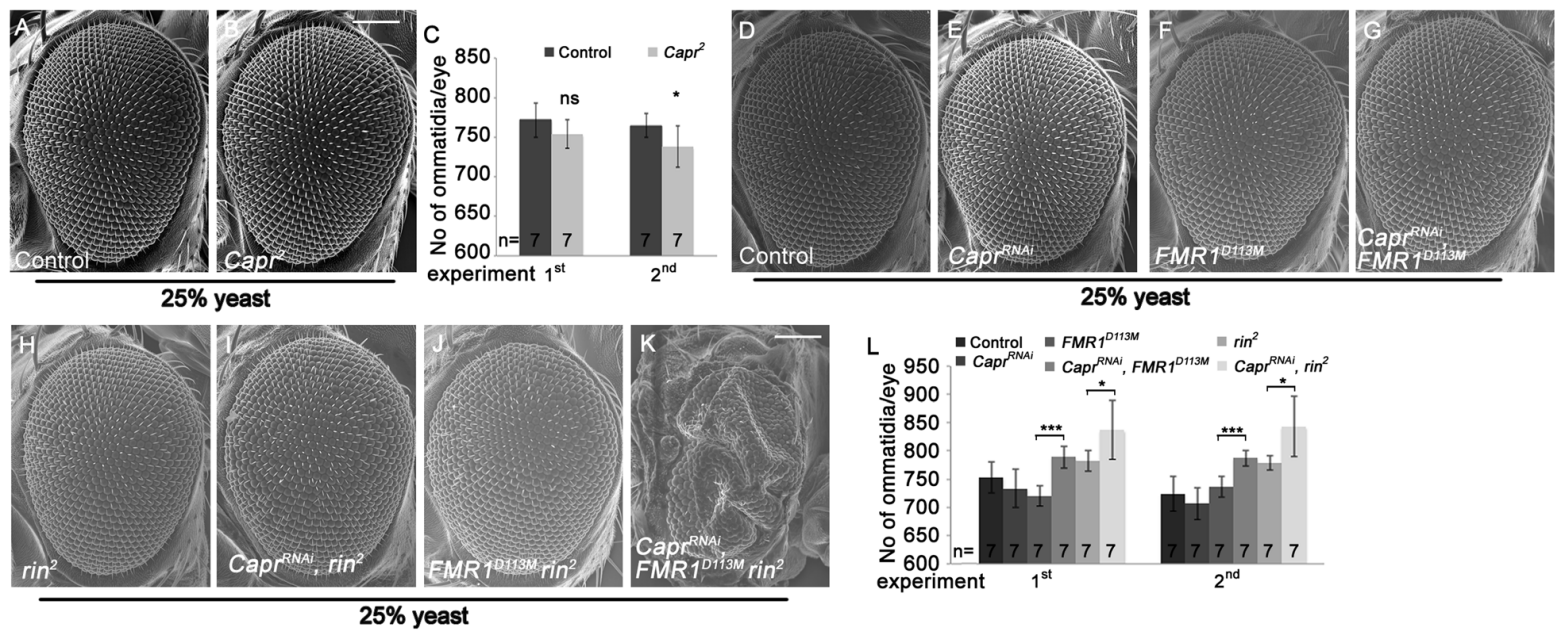
Capr cooperates with FMR1 and Rin to suppress growth. (A–B) Scanning electron micrographs of adult control (A) and *Capr^2^* (B) eyes generated by eyFLP/FRT Minute mediated mitotic recombination from flies reared on 25% yeast food (A–B). Scale bar represents 100 µm. (C) Statistical analyses as described in Figure 1D: control (772±22 and 765±15) and *Capr^2^* (754±18; p = 0.11 and 738±26; p = 0.04) mutant eyes from flies raised on 25% yeast-containing food. (D–K) Scanning electron micrographs of adult control (D), *Capr^RNAi^* (E), *FMR1^D113M^* (F), *Capr^RNAi^ FMR1^D113M^* (G), *rin^2^* (H), *Capr^RNAi^ rin^2^* (I), *FMR1^D113M^ rin^2^* (J) and *Capr^RNAi^ FMR1^D113M^ rin^2^* eyes (K) generated by eyFLP, Actin-Flp out-Gal4/FRT-mediated mitotic recombination from flies grown on 25% yeast-containing food. Scale bar represents 100 µm. (L) Statistical analyses as described in Figure 1D: control (753±28 and 724±31), *Capr^RNAi^* expressing (733±34 and 706±28), *FMR1^D113M^* mutant (720±18 and 736±19), *Capr^RNAi^* expressing *FMR1^D113M^* mutant (789±20; p = 1.82E-05 and 787±14; p = 0.00015), *rin^2^* mutant (782±18 and 779±13) and *Capr^RNAi^* expressing *rin^2^* mutant (837±52; p = 0.033 and 843±53; p = 0.018) eyes from flies raised on 25% yeast-containing food. Genotypes: (A) *y w eyFLP*/*y w*; *M(3)RpS17^4^ FRT80*/*FRT80* (B) *y w eyFLP*/*w*; *M(3)RpS17^4^ FRT80*/*Capr^2^ FRT80* (D) *y w eyFLP*, *Act>CD2>Gal4*/*y w*; *FRT82 cl w^+^*/*FRT82* (E) *y w eyFLP*, *Act>CD2>Gal4*/*y w*; *UAS-Capr^RNAi^*/+; *FRT82 cl w^+^*/+ (F) *y w eyFLP*, *Act>CD2>Gal4*/*y w*; *FRT82 cl w^+^*/*FRT82 FMR1^D113M^* (G) *y w eyFLP*, *Act>CD2>Gal4*/*y w*; *UAS-Capr^RNAi^*/+; *FRT82 cl w^+^*/*FRT82 FMR1^D113M^* (H) *y w eyFLP*, *Act>CD2>Gal4*/*y w*; *FRT82 cl w^+^*/*FRT82 rin^2^* (I) *y w eyFLP*, *Act>CD2>Gal4*/*y w*; *UAS-Capr^RNAi^*/+; *FRT82 cl w^+^*/*FRT82 rin^2^* (J) *y w eyFLP*, *Act>CD2>Gal4*/*y w*; *FRT82 cl w^+^*/*FRT82 FMR1^D113M^ rin^2^* (K) *y w eyFLP*, *Act>CD2>Gal4*/*y w*; *UAS-Capr^RNAi^*/+; *FRT82 cl w^+^*/*FRT82 FMR1^D113M^ rin^2^*.

Recently, *Capr^2^* null mutants were described to be viable without morphological alterations, and Capr and FMR1 cooperatively regulate the cell cycle at the mid-blastula transition [9]. We wondered whether Capr acts redundantly with FMR1 and Rin in growth control in epithelial tissues. To characterize the *Capr* growth phenotype, we generated mutant eyes during development using the eyFLP/FRT technique or by downregulation of *Capr* via RNAi. Note that we used a *Minute* mutation instead of a cell lethal mutation on the FRT80 chromosome. *Capr^RNAi^* overexpression in clones resulted in a strong reduction of Capr protein (Figure S6A'), proving the functionality of the RNAi line. Both *Capr^2^* null mutant eyes and eyes with downregulated *Capr* displayed slightly reduced eye size in comparison to the controls (Figure 6B, 6C, 6E and 6L). In contrast, downregulation of *Capr* in *FMR1* or *rin* mutant eyes resulted in overgrown eyes due to more ommatidia (Figure 6G, 6I and 6L). Furthermore, downregulation of *Capr* in *FMR1 rin* double mutant eyes resulted in late pupal lethality. Analysis of eyes and heads revealed strongly overgrown structures in pharate adults (Figure 6K), suggesting that FMR1, Rin and Capr act synergistically in growth regulation.

### Lig synergizes with FMR1, Rin and Capr and controls *rin* expression at the transcriptional level

**Figure 7:**
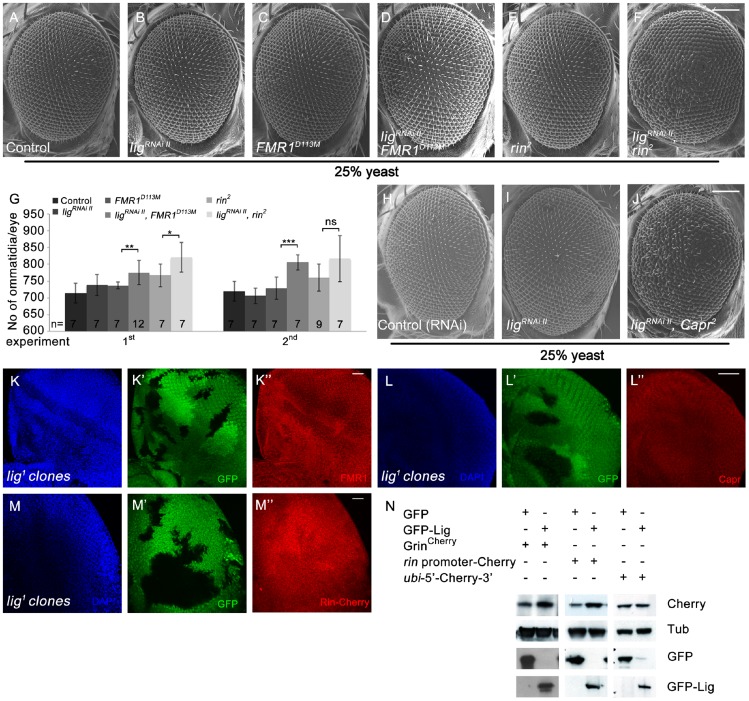
Lig cooperates with FMR1, Rin and Capr in growth control and regulates *rin* at the transcriptional level. (A–G) Scanning electron micrographs of adult control (A), *lig^RNAi II^* (B), *FMR1^D113M^* (C), *lig^RNAi II^ FMR1^D113M^* (D), *rin^2^* (E), *lig^RNAi II^ rin^2^* (F) eyes generated by eyFLP Actin-Flp out-Gal4/FRT-mediated mitotic recombination from flies grown at 25% yeast content (A–F). Scale bar represents 100 µm. (G) Statistical analyses as described in Figure 1D: control (714±30 and 720±30), *lig^RNAi II^* expressing (738±32 and 706±23), *FMR1^D113M^* mutant (737±10 and 729±34), *lig^RNAi II^* expressing *FMR1^D113M^* mutant (775±36; p = 0.0051 and 806±23; p = 0.00046), *rin^2^* mutant (767±34 and 760±40), *lig^RNAi II^* expressing *rin^2^* mutant (821±45; p = 0.03 and 817±69; p = 0.082) eyes from flies raised on 25% yeast-containing food. (H–J) Scanning electron micrographs of adult control (H), *lig^RNAi II^* (I) and *lig^RNAi II^ Capr^2^* (J) eyes generated by eyFLP Actin-Flp out-Gal4/FRT Minute-mediated mitotic recombination from flies grown on 25% yeast-containing food. (K–M'') Negatively marked 72 h old *lig^1^* mutant clones (induced with the hsFLP/FRT system) in eye imaginal discs of third instar larvae (K', L' and M'). FMR1 levels (visualized by immunostaining) remain unchanged (K''). Capr levels (visualized by immunostaining) are slightly increased in *lig* mutant clones (L''). Rin-Cherry levels expressed from the *Grin^Cherry^* transgene are autonomously decreased in the *lig^1^* mutant clones (M''). Imaginal discs are stained with DAPI (blue) to visualize the DNA. Scale bar represents 25 µm. (N) S2 cells transfected with GFP-Lig and Grin^Cherry^ have increased levels of Rin-Cherry in comparison to S2 cells overexpressing GFP and Grin^Cherry^. S2 cells overexpressing GFP-Lig upregulate a transcriptional reporter consisting of the *rin* promoter followed by a *Cherry* coding sequence and the 3′ UTR of *rin*. Conversely, a translational reporter consisting of an *ubi* promoter followed by the 5′ UTR of *rin*, a *Cherry* protein coding sequence and the 3′ UTR of *rin* is not affected. Genotypes: (A) *y w eyFLP*, *Act>CD2>Gal4*/*y w*; *FRT82 cl w^+^*/*FRT82* (B) *y w eyFLP*, *Act>CD2>Gal4*/*y w*; *UAS-lig^RNAi II^* [51D]/+; *FRT82 cl w^+^*/+ (C) *y w eyFLP*, *Act>CD2>Gal4*/*y w*; *FRT82 cl w^+^*/*FRT82 FMR1^D113M^* (D) *y w eyFLP*, *Act>CD2>Gal4*/*y w*; *UAS-lig^RNAi II^* [51D]/+; *FRT82 cl w^+^*/*FRT82 FMR1^D113M^* (E) *y w eyFLP*, *Act>CD2>Gal4*/*y w*; *FRT82 cl w^+^*/*FRT82 rin^2^* (F) *y w eyFLP*, *Act>CD2>Gal4*/*y w*; *UAS-lig^RNAi II^* [51D]/+; *FRT82 cl w^+^*/*FRT82 rin^2^* (H) *y w eyFLP*, *Act>CD2>Gal4*/*y w*; *UAS-CG1315^RNAi^* (control)/+; *M(3)RpS17^4^ FRT80*/+ (I) *y w eyFLP*, *Act>CD2>Gal4*/*y w*; *UAS-lig^RNAi II^* [51D]/+; *M(3)RpS17^4^ FRT80*/+ (J) *y w eyFLP*, *Act>CD2>Gal4*/*y w*; *UAS-lig^RNAi II^* [51D]/+; *M(3)RpS17^4^ FRT80*/*Capr^2^ FRT80* (K, L) *y w hsFLP*/*y w*; *FRT42 ubiGFP*/*FRT42 lig^1^* (M) *y w hsFLP*/*y w*; *FRT42 ubiGFP*/*FRT42 lig^1^*; *Grin^Cherry^* [86Fb]/+.

The similarity of the *lig* and the *FMR1*, *rin* or *Capr* phenotypes in combination of double mutants prompted us to genetically test whether Lig regulates growth via FMR1, Rin and Capr. We downregulated *lig* via RNAi in *FMR1*, *rin* or *Capr* mutant eyes induced by the eyFLP/FRT system. Note that *lig* RNAi eyes did not consist of more ommatida under reduced food conditions (Figure 7B and 7H) in comparison to flies raised under normal conditions (Figure S1W). Reduced Lig levels in *FMR1* or *rin* mutant eyes increased the eye size due to more ommatidia (Figure 7D, 7F and 7H). Flies with *Capr* mutant eyes and reduced *lig* were dying as pharate adults with increased and disturbed eye structures (Figure 7J). We conclude that Lig cooperates with FMR1, Rin and Capr in growth control. The fact that the single mutants of *FMR1*, *rin* or *Capr* have no or minor effects on growth regulation, whereas the double mutants have similar effects like *lig* mutants, suggests that Lig modulates FMR1, Rin and Capr function in concert.

Next we checked the localization and protein levels of FMR1, Capr and Rin in *lig* mutant clones induced by the hsFLP/FRT system in developing eyes. Whereas FMR1 showed no localization or abundance alterations (Figure 7K'') and Capr only a slight upregulation (Figure 7L'') in *lig* mutant cells, Rin-Cherry levels were reduced in *lig* mutant clones (Figure 7M''), indicating that Lig mainly regulates Rin levels.

Vice versa, Rin-Cherry levels were upregulated in *lig* overexpressing clones in eye imaginal discs (Figure S7A' and S7A''). Recently, Rin has been identified as substrate for ubiquitination in the central nervous system [26]. To test whether Lig regulates Rin at the protein level, we induced *lig* null mutant clones in eye imaginal discs expressing a HA-tagged Rin under the control of an UAS promoter. In this situation, Lig was not able to regulate Rin (Figure S7B' and S7B''), excluding Lig as stabilizer of the Rin protein. We then investigated whether Lig regulates *rin* at the transcriptional and/or translational level. Lig overexpression in S2 cells was able to increase Rin-Cherry expressed by *Grin^Cherry^* (Figure 7N). To generate a translational reporter, we placed the 5′ and 3′ UTRs of *rin* mRNA upstream and downstream of a Cherry-coding region under control of the *ubi* promoter. The transcriptional reporter expressed the Cherry-coding sequence and the 3′UTR of *rin* under control of the *rin* promoter. Co-expression of *lig* with the transcriptional reporter construct, but not with the translational reporter, was able to increase Cherry levels, suggesting that Lig impacts on *rin* transcription (Figure 7N).

### Lig regulates a reporter of the JAK/STAT pathway

**Figure 8:**
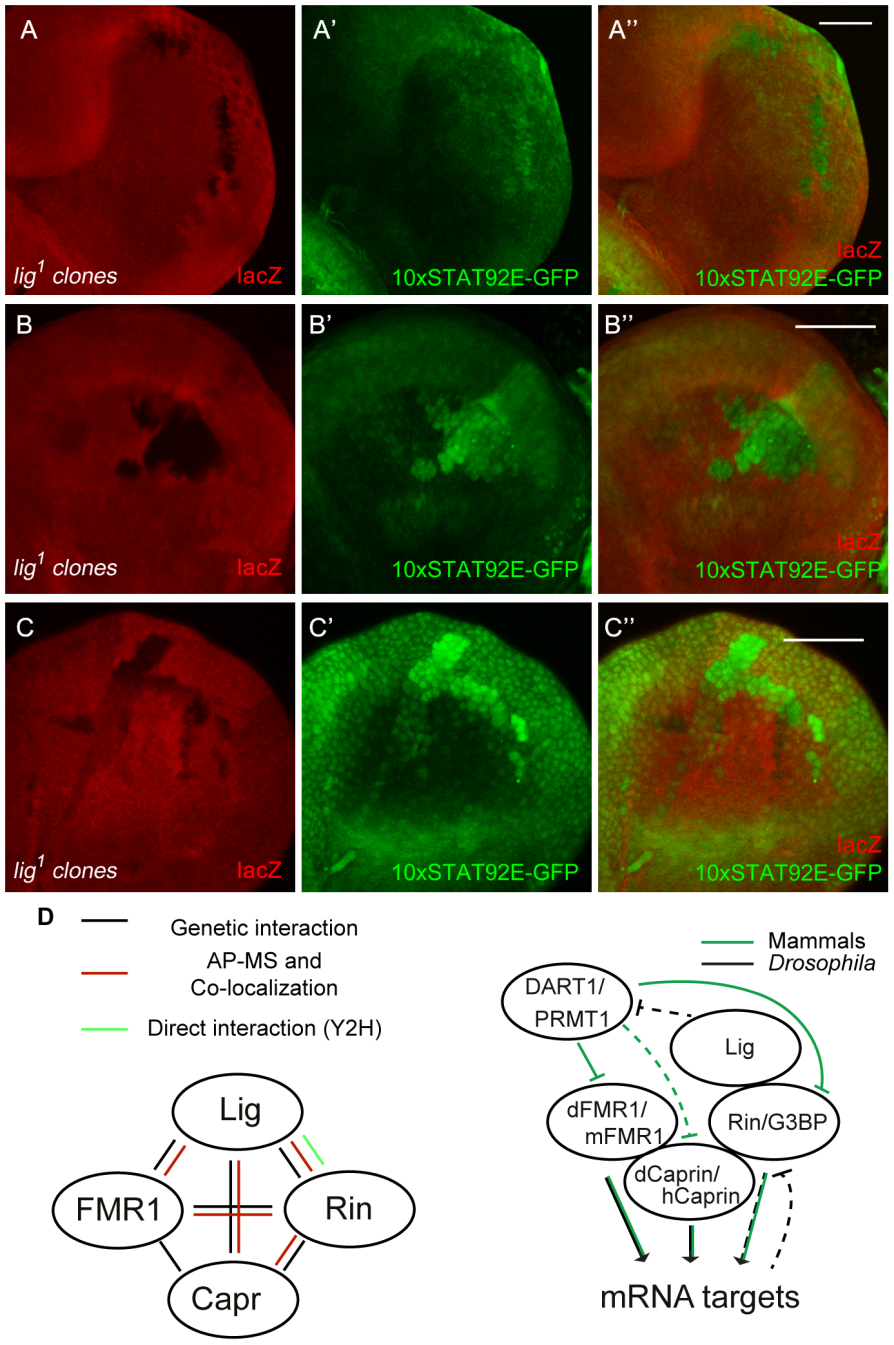
JAK/STAT signaling is activated in *lig* mutant cells. (A–C) *lig^1^* mutant clones (induced with the FLP/FRT system, 72 h old, marked by the lack of lacZ staining) in eye (A and A''), antenna (B and B'') and wing (C and C'') imaginal discs of early third instar larvae. The JAK/STAT signaling reporter 10xSTAT92E-GFP is upregulated in *lig1* mutant clones in the posterior side of the eye imaginal disc (A' and A''), antenna imaginal disc (B' and B'') and in the hinge region of the wing disc (C' and C''). Note that the reporter signal is autonomously increased in the mutant clones. Scale bars represent 50 µm. (D) Schematic representation of the interactions shown in this study (left) and a working model of a Lig/Rin/FMR1/Caprin complex (right). Genotypes: (A–C) *y w hsFLP*/*y w*; *FRT42 arm-lacZ*/*FRT42 lig^1^*; *10xSTAT92E-GFP*/+.

We demonstrated that Lig regulates cell proliferation in concert with the mRNA binding proteins FMR1, Rin and Capr. To investigate which growth signaling pathway is altered, we tested readouts for various signaling pathways in *lig* mutant clones in wing and eye imaginal discs. FMR1 binds to the miRNA *bantam* to control the fate of germline stem cells [6]. *bantam* miRNA is a known target of the Hippo signaling pathway [27], [28] and inhibits the pro-apoptotic gene *hid* [29]. If Lig regulates the Hippo pathway and/or *bantam* miRNA, we would expect an upregulation of a minimal Hippo response element (DIAP4.3-GFP) and downregulation of the bantam sensor. In both experiments we did not observe any alteration of the reporter signal (Figure S8A'', S8B''). Consistently, overexpression of *lig* did not upregulate the bantam sensor (S8C''). Furthermore, FMR1 was reported to regulate *cbl* mRNA, a negative regulator of the EGFR, to control germline cell proliferation in ovaries [30]. However, a transcriptional reporter for *pointed* expression, a target of the EGFR pathway, was not changed in *lig* mutant clones in eye imaginal discs (Figure S8D''). Recently, increased Insulin signaling has been observed in *FMR1* mutant brains using pAkt as readout [31]. In *lig* mutant clones in eye imaginal discs, we observed neither an increase of pAkt nor a recruitment of pAkt to the membrane, a sign for active Insulin signaling (Figure S8E''). The Rin ortholog G3BP is involved in human *c-myc* mRNA decay by an intrinsic endonuclease activity [12], [13]. However, we did not detect any alterations of Myc levels in *lig* mutant clones (Figure S8F''). Recently, it was demonstrated that G3BP is involved in Wnt/β-catenin signaling by binding and regulation of *β-catenin* mRNA [5]. To test an involvement of Lig via Rin in Wnt signaling, we stained imaginal discs harboring *lig* mutant clones for Distal-less (Dll) and Senseless (Sens), two target genes of the Wnt signaling pathway in *Drosophila*. We did not observe any alterations of the Dll expression pattern in wing imaginal discs (Figure S8G'') or of the Sens expression patterns in wing (Figure S8H'') and eye imaginal discs (Figure S8I''), arguing against an involvement of Lig in Wnt signaling. We also tested Hedgehog, Notch and JAK/STAT signaling. Whereas Ptc and Cut patterns, targets of the Hedgehog and Notch signaling pathway, respectively, were not altered in *lig* mutant clones (Figure S8J'' and S8K''), a JAK/STAT reporter (10xSTAT92E-GFP) was upregulated in *lig* mutant clones. GFP expression from the 10xSTAT92E-GFP reporter was autonomously increased in *lig* mutant clones in the posterior region of eye discs (Figure 8A''), in antenna discs (Figure 8B'') and in the pleura and hinge regions of wing discs (Figure 8C'') of early third instar larvae. Consistent with our findings, Lig was identified as negative regulator of JAK/STAT signaling in an RNAi based screen in cultured *Drosophila* Kc cells [32]. To determine whether Lig has an effect on STAT92E protein levels, we analyzed STAT92E expression in *lig* mutant clones in eye imaginal discs. We did not observe any alteration of STAT92E levels in the posterior region but an upregulation of STAT92E in the anterior region of the eye imaginal disc (Figure S8L''). Thus, based on the autonomous effects on the 10xSTAT92E-GFP reporter and on STAT92E levels, Lig regulates intracellular components of the JAK/STAT signaling pathway rather than the ligands.

## Discussion

We have identified Lig as a new growth suppressor in eye and wing epithelial tissues. Whereas eyes mutant for *lig* consist of more ommatidia without cell size defects, eyes overexpressing *lig* have a reduced cell number due to increased apoptosis and reduced cell cycle progression. *lig* mutant eyes are sensitive to apoptosis (resulting in a variable phenotype under normal food conditions) but are able to cope with the overgrowth situation when the flies develop under suboptimal growth conditions. Similarly, the reduced eye phenotype of *lig* overexpressing eyes was partially rescued under suboptimal growth conditions or by expression of DIAP1, suggesting that the starvation response impacts on the apoptosis rates in imaginal discs. However, we cannot exclude other indirect effects that might be triggered by starvation.

In addition to our findings, *lig* mutants have previously been characterized for their behavioral phenotype in the copulation process [15] and their putative role in neuronal tissues [33]. Lig is conserved from flies to humans, the human orthologs being ubiquitin associated protein 2 (UBAP2) and ubiquitin associated protein 2 like (UBAP2L). UBAP2 has been identified in a Y2H screen as a direct interaction partner of the zona pellucida 3 (ZP3) protein that is involved in sperm binding and acrosomal exocytosis [34]. UBAP2L has been reported to accumulate at ubiquitin-rich aggregates upon proteasome inhibition in human neuroblastoma tissue culture cells, suggesting that the UBA domain is functional [35]. It is currently unknown whether the Lig orthologs are involved in growth regulation, and no interaction partners have been identified except for ZP3.

Several lines of evidence indicate that Lig interacts with FMR1, Capr and Rin, and via these interactions functions to regulate growth: (i) Lig associated with FMR1 and Rin in an AP-MS experiment, (ii) Lig co-localized with FMR1, Capr and Rin, (iii) Lig directly interacted with Rin in a Y2H experiment, (iv) Lig transcriptionally regulated Rin levels, and (v) *FMR1*, *Capr* or *rin* in combination of double mutants behaved like *lig* null mutants and (vi) *lig* downregulation in *FMR1*, *Capr* or *rin* mutant eyes synergistically increased the eye size (Figure 8D).

The interaction between Lig and Rin, Capr and FMR1, three RNA-binding proteins, and the co-localization with P-body components suggests that Lig regulates the translation and/or stability of specific mRNAs of growth-regulatory genes via FMR1, Capr and Rin function. Indeed, the *Drosophila* FMR1 and orthologs of Rin are involved in translational regulation of growth-regulatory genes in certain tissues. For example, FMR1 binds *bantam* miRNA, an inhibitor of the pro-apoptotic gene *hid* [29], and regulates *cbl*, which encodes a component of the EGFR signaling pathway, in germline stem cells [30]. However, *bantam* miRNA is not regulated by FMR1 in epithelial cells [36], and Lig was unable to regulate a *bantam* miRNA reporter. Furthermore, the expression of a *pointed* transcriptional reporter was unchanged in *lig* mutant clones, suggesting that *cbl* regulation by FMR1 is specific to the germline or has only subtle effects in the developing eye. The Rin ortholog G3BP controls *myc* [12], [13], *CyclinD2* [4], *cdk7* and *cdk9* mRNA [3]. However, it is not known whether this function is conserved for Rin, and we did not observe any alterations of Myc protein levels in *lig* mutant clones. It will be important to identify mRNAs that are regulated by FMR1, Capr and Rin in epithelial tissues during development, and to determine whether Lig mediates specificity for certain mRNAs.

To identify the signaling pathway that is regulated by Lig, we used readouts for the Hippo, EGFR, Insulin, Hedgehog, Wnt and JAK/STAT signaling pathways. We observed no alterations of all analyzed pathways except for the highly conserved JAK/STAT signaling pathway. The pathway is composed of four modules: the ligands, Upd, Upd2 and Upd3, the receptor Domeless (Dome), the receptor-associated Janus kinase (JAK) Hopscotch (Hop), and the signal transducer and activator of transcription (STAT) STAT92E (reviewed in [37]). The involvement of Lig in the JAK/STAT signaling pathway leads to a number of assumptions and questions in the context of our findings. First, the autonomous effect of Lig on the 10xSTAT92E-GFP reporter suggests that Lig regulates the intracellular components (Dome, Hop or STAT92E) or modifiers thereof rather than expression of the ligands, which would result in non-autonomous effects. Second, the physical and genetic interactions of Lig with the mRNA binding proteins FMR1, Capr and Rin raises the question whether Lig directly impacts on the JAK/STAT pathway or whether it modulates the JAK/STAT signaling via FMR1, Capr and Rin. So far, we cannot exclude either option. However, it was recently demonstrated that *upd* and *STAT92E* mRNAs are targets for posttranscriptional regulation via the miRNA pathway [38], [39]. It will be interesting to determine whether FMR1, Rin or Capr are involved in this process in the case of STAT92E.

Our data provide evidence that FMR1, Capr and Rin function in a redundant manner in epithelial tissues in growth control, suggesting that they regulate either overlapping sets of mRNAs or different mRNAs encoding proteins with redundant functions. Examples for the former have been described for FMR1, Capr and G3BP, the human ortholog of Rin. In *Drosophila*, FMR1 cooperates with Capr, and both proteins bind to the same mRNAs *frs* and *CycB* [9]. Similarly, G3BP forms a complex with human Caprin and both interact with *myc* and *CycD* mRNAs [4]. Both examples suggest a redundant regulation of these targets. There is no direct evidence for the latter possibility. However, G3BP associates with and translationally regulates *tau* mRNA in neuronal cells [40], [41]. In *Drosophila*, FMR1 negatively regulates *futsch* mRNA [42], and the *futsch* mutant phenotype is suppressed by overexpression of *Tau* [43], suggesting a redundant function of Tau and Futsch.

Lig impacts on Rin and slightly on Capr but not on FMR1 levels. However, only *FMR1*, *Capr* or *rin* mutants in combination as double mutants resulted in a *lig* like phenotype, suggesting that the activity of FMR1 and Capr is altered (probably at the posttranslational level) in a *lig* mutant situation. Our AP-MS experiments also revealed DART1 as a physical binding partner of Lig. Arginine methyl transferases are able to methylate RGG motifs and thereby modulate the binding capability to mRNAs [44], [45]. Interestingly, FMR1 contains a conserved RGG domain that can be methylated in *Drosophila* and humans. In humans, protein methyl transferase 1 (PRMT1), the ortholog of DART1, mediates the arginine methylation of FMR1 to alter its binding affinity to mRNAs [46] (Figure 8D). Furthermore, G3BP1, the mouse ortholog of Rin, contains an RGG domain that is methylated by PRMT1 after stimulation of the Wnt signaling pathway to modulate the binding to *β-Catenin* mRNA [5]. The RGG domain of Rin is weakly conserved and lacks the RGG motifs. It is thus unclear whether Rin can be methylated in the truncated arginine-glycine rich region. Like FMR1 and G3BP, Caprin contains RGG domains, and it was identified as binding partner of PRMT8, which is closely related to PRMT1 at the sequence level [47]. Further experiments are required to resolve whether Lig is involved in a DART1-mediated methylation of FMR1 and Rin under certain conditions, or whether Lig alters the activity of FMR1 and Capr by another mechanism.

Lig, FMR1, Rin and Capr have been identified as interactors of Orb in Co-IP experiments [18], suggesting a complex formation of these proteins. Complex formation has been reported for G3BP and Caprin in human cell lines [4] and for Capr and FMR1 in *Drosophila* [9] and mouse neurons [8] so far (Figure 8D). We were able to demonstrate that Rin, Capr and FMR1 have a redundant function in the eye, and that they localize in the same subcellular structure in cultured *Drosophila* cells. This raises the question whether the three RNA-binding proteins Capr, Rin and FMR1 are functionally related only in the eye. Systematic analyses of the phenotypes of double mutant combinations will reveal the tissues in which these RNA-binding proteins exert redundant and non-redundant functions. Furthermore, it will be interesting to determine whether Rin and Capr contribute to phenotypes associated with the FXS.

## Materials and Methods

### Fly stocks and culture conditions

EMS-induced *lig* mutant alleles were recovered in an unbiased eyFLP/FRT cell lethal screen [14]. *lig^1^* harbors a small deletion of 5 bp (nucleotides 3959163–3959167) and an insertion of an adenine at position 3959174. *lig^2^* includes a small deletion of 17 bp (nucleotides 3958424–3958440). The nucleotide positions are based on the release 5.45 of the *Drosophila* genome. *lig^3^* contains a point mutation changing W155 into a stop codon. The following *FMR1*, *rin* and *Capr* alleles and transgenes were used: *lig^PP1^* [15], *Df(2R)Exel7094* (BDSC no. 7859), *Glig* [61B3] [15], *Glig^FS^* [86Fb], *FMR1^D113M^* (BDSC no 6929, [42]), *FMR1^D50M^* (BDSC no 6930, [42]), *rin^2^* (BDSC no 9303, [48]), *P{GawB}rin^NP3248^* (DGRC no 104425), *P{GawB}rin^NP5420^* (DGRC no 113726), *Capr^2^* [9], *UAS-Capr^RNAi^* (VDRC 110272). The alleles *FMR1^D113M^* and *FMR1^D50M^*, *rin^2^*, *P{GawB}rin^NP3248^*, *P{GawB}rin^NP5420^* were recombined onto FRT82. The presence of *FMR1^D113M^* and *FMR1^D50M^* as well as of *rin^2^* deletions was verified by PCR using the primer pairs FMR1_F, FMR1_R and Rin_F, Rin_R, respectively. Sequencing of the PCR product generated with the primer pair Rin_F, Rin_R revealed the break points of the *rin^2^* deficiency at positions 9473220 and 9486306.

The eyFLP/FRT-cell lethal recombination system [49] or eyFLP/FRT M was used to generate mutant heads. To express *UAS* transgenes in clones in eye and wing imaginal discs, the Actin-Flp out-Gal4 technique was used [50]. Clones were induced in second instar larvae (heat shock for 10 min at 37°C, 48 hours before dissection), and the imaginal discs were dissected from third instar larvae. Negatively marked mutant clones were generated with the hsFLP/FRT-ubiGFP system. Clones were induced in first instar larvae (heat shock for 15 min at 37°C, 72 hours before dissection), and the eye imaginal discs were dissected from third instar larvae.

Additional fly strains used in this study were: *nubbin-Gal4* [51], *da-Gal4* (BDSC), *DE-Gal4* [52], *ey-Gal4* (insertion on 2nd chromosome) [53], *UAS-CycE* [54], *EP-Diap1* (BDSC), *P{Fmr1.14}* [55], *UAS-p35* (BDSC), *DIAP1-GFP4.3* [56], *10xSTAT92E-GFP* [57], MIR33 bantam sensor (gift from Stephen Cohen), *pnt-lacZ* (*P{lacW}pntS0998*, former stock collection of Szeged, No. 121625).

Genetic experiments were conducted at 25°C. Food with 100% yeast consists of 7.5 g sugar, 5.5 g corn, 1 g flour, 0.8 g Agar, 1.5 ml Nipagin/Nipasol and 10 g fresh yeast filled up to 100 ml with tap water. For fly food with 25% or 40% yeast, the yeast amount was reduced to 2.5 g and 4 g yeast, respectively. 3.3 g Casein was used to substitute 40% yeast-containing food to 100% amino acid-containing food. For fly food with 400% yeast, the yeast amount was 40 g fresh yeast. 10 ml of food was filled into vials with a diameter of 29 mm. For experiments with different food conditions, 100–150 embryos of each cross were collected from apple agar plates and distributed to individual vials.

### Analysis of adult flies

To assess the ommatidia number, flies were exposed to dimethyl ether for 7–10 min before taking scanning electron micrographs with a JEOL 6360 VP microscope. The ommatidia number was counted using a semi-automated ommatidia counter software (Ommatidia counter, version 0.3, programmed by Vasco Medici, SciTrackS GmbH). Pictures from pupae and adult wings were taken with a Keyence VHX-1000 microscope. Tangential eye sections of adult eyes were done as previously described [58].

### Cloning and generation of transgenic fly lines

The *Glig* was subcloned from *pCaSpeR-Glig* [15] into the gattb vector using the restriction sites XhoI and XbaI. The frameshift in the *Glig^FS^* construct was obtained as a spontaneous mutation during the subcloning.

For the *lig* RNAi lines, the regions I (308 bp) and II (252 bp) were amplified with the primer pairs Lig_RNAi_FB, Lig_RNAi_RB and Lig_RNAi_FC, Lig_RNAi_RC, respectively, using pENTR-lig as template. The fragments were first digested with EcoRI and then self-ligated. The resulting inverted repeats were cloned into a modified gattb vector, attB-genxpMF3. attB-genxpMF3 was generated by cloning a fragment of the pMF3 vector containing the promoter, restriction sites for subcloning of the hairpin and the polyA signal, into the gattb vector using the restriction sites NotI and BamHI.

The *lig^R185C/UTR^* sequence was subcloned from pUAST-lig^R185C/UTR^ (gift from Yamamoto lab) into the pUAST attb vector using the restriction site EcoRI. The *lig* coding region sequence was amplified from pUAST-lig^R185C^ and cloned into the pENTR vector. Site-directed mutagenesis was used to obtain the *lig* coding region without the C553T substitution that causes the amino acid exchange R185C. Analysis of *UAS-lig^R185C^* revealed similar phenotypes as observed for *UAS-lig* (Figure S2B and S2C), suggesting that the amino acid exchange R185C represents a polymorphism. pENTR-lig^FG-LA^ was generated by site-directed mutagenesis with the primers LigF_LA and LigR_LA using pENTR lig as template.

The coding sequence of *rin* was cloned into pENTR. LR reaction was used to subclone the coding sequences from pENTR-lig and pENTR-rin into the Gateway vectors pUAST-W-attb and pUAST-HW-attb.

The gattB-Grin and gattB-Grin^Cherry^ vectors were cloned in two and three steps, respectively. A fragment of 7.2 kbp from the P[acman] BAC 13D12 [59] was subcloned into a modified gattb vector using BamHI and AgeI restrictions sites. In the second step, a PCR-amplified fragment of 4.6 kbp (using the primer pair Rin_FA, gRin_R) was subcloned into the gattb vector containing the 7.2 kbp Grin fragment using the restriction sites AgeI and NotI, resulting in the construct gattB-Grin. A *cherry* coding sequence including a stop codon was fused to the third exon of *rin* without stop codon and to the 3′ UTR of *rin* by fusion PCR.

Transgenic flies were generated with the site-specific phiC31 integration system using vas-φC31-zh2A; ZH-attP-44F, vas-φC31-zh2A; ZH-attP-51D and vas-φC31-zh2A; ZH-attP-86Fb embryos [60].

### Cell culture, transfection, Western blot and AP-MS

S2 cells were cultured and transfected according to standard protocols.

The coding sequences of *FMR1*, *Capr* and *DART1* were cloned into pENTR. LR reactions were performed to subclone the coding sequences from pENTR-GFP, pENTR-FMR1, pENTR-Capr, pENTR-DART1, pENTR-rin, pENTR-lig, pENTR-lig^R185C^ into the Gateway vectors pMHW, pAGW, pARW and pAFW. GFP-DCP1 was used as a P-body marker [61].

For the *rin* translational reporter construct, the two parts of the 5′ UTR of *rin* were amplified with the primer pairs EcoRI_Rin_F, Rin_RA and Rin_FB, NotI_Rin_R, respectively, from genomic DNA of *y w* flies, fused by fusion PCR and subcloned into the gattb vector containing an *ubi* promoter using the restriction sites EcoRI and NotI. The coding sequence of *cherry* fused to the 3′ UTR of *rin* was amplified with the primer pair NotI_Cherry_F, XbaI_Rin_R from the template gattB-RinCherry and subcloned into the gattb-ubi-5′ UTR *rin* vector using the restriction sites NotI and XbaI.

For the *rin* transcriptional reporter, the ubi-5′ UTR of *rin* of the translational reporter was replaced with the *rin* promoter that was amplified with the primer pair gattB_F, Rin_RG from the template gattB-Grin^Cherry^.

Western blots were performed according to standard protocols. AP-MS analysis was done as described in [62].

### Primers used in this study

RinF, 5′-CACCATGGTCATGGATGCGACCC-3′; RinR, 5′-GCGACGTCCGTAGTTGCC-3′; FMR1_FB, 5′-CACCATGGAAGATCTCCTCGTG-3′; FMR1_RB, 5′-GGACGTGCCATTGACCAG-3′; Lig_RNAi_I_F, 5′-GAGAATTCCAGCAGCAGACGACGCCTATCA-3′; Lig_RNAi_I_R, 5′-CATCTAGATTCGAGGGTGGTGGCAGCTT-3′; Lig_RNAi_II_F, 5′-GAGAATTCCCACAAATACCGGCAGCAAACA-3′; Lig_RNAi_II_R, 5′-CATCTAGAGTTAAACGGGGGCGGAGTGC-3′; LigF_LA, 5′-GGACGTGCAGTTaGcCGCTCTGGACTTaGcCACGGACGATGG-3′; LigR_LA, 5′- CCATCGTCCGTGgCtAAGTCCAGAGCGgCtAACTGCACGTCC-3′; EcoRI_Rin_F, 5′-TAGAATTCATCATTCACACACCAACACACG-3′; Rin_RA, 5′-CCTAGACGACTGTGTAGCTTTTTTAAGCGATATTTTTCCTCG-3′; Rin_FB, 5′-CGCTTAAAAAAGCTACACAGTCGTCTAGGACTTTTGC-3′; NotI_Rin_R, 5′-ATCGCGGCCGCAGCTGGCGTTTGATTCTTCCTC-3′; NotI_Cherry_F, 5′-TAGCGGCCGCGATGGTGAGCAAGGGCGAGGAGG-3′; XbaI_Rin_R, 5′-ATTCTAGAGTTGCTTGACTTAGTTTGGTTTACG-3′; gattB_F, 5′-GAAAATGCTTGGATTTCACTGG-3′; Rin_FA, 5′-GGTGGCACCACAGCTCAT-3′; gRin_R, 5′-TAGCGGCCGCCAGGCGATTCCGTTCAAGATATTTAATAAATAATAAAG-3′.

### Antibody stainings

S2 cells or eye imaginal discs were fixed in 4% PFA at RT for 20 min and blocked with 2% NDS in 0.3% PBT or 1% BSA in 0.3% PBT (only for rabbit α-Cleaved Caspase-3 antibody). The following primary and secondary antibodies were used: mouse α-Ago1 (1∶300; [63]), rabbit α-Cleaved Caspase-3 (1∶300, Cell signaling, Catalog no. 9661), mouse α-Lig-N (1∶300) [15], mouse α-FMR1 clone 6A15 (1∶300) [64], rabbit α-Capr (1∶1000) [9], mouse α-FLAG (Sigma, F1804), mouse α-HA (Convance, MMS-101R), mouse α-GFP (Roche, 11814460001), mouse α-mCherry (Abcam, ab125096), rabbit α-pAkt (Ser 473) (1∶300, Cell signaling, 9277S), rabbit α-Myc (1∶5000) [65], mouse α-Dll (Ian Duncan; gift from K. Basler), guinea pig α-Sens (GP55, 1∶800, H. Bellen, Baylor College of Medicine, Houston; gift from K. Basler), mouse α-Ptc (1∶100, DSHB), mouse α-Cut 2B10 (1∶100, DSHB), rabbit α-STAT92E (1∶1000) [66], goat α-rabbit Cy3 (GE Healthcare, PA43004), goat α-mouse Cy3 (GE Healthcare PA43002), α-mouse Cy5 (GE Healthcare, PA45002), α-mouse HRP (Jackson ImmunoResearch, 115-035-003).

Pictures were taken using a Leica SPE or SP2 confocal laser scanning microscope.

### Yeast two-hybrid assay

Yeast two-hybrid analysis was carried out using Invitrogen's ProQuest Two-Hybrid System with Gateway Technology according to the manufacturer's instructions. Full- length cDNAs and the cDNA fragments of *lig*, *FMR1*, *Capr*, and *rin, and lig^256–1333^*, *lig^FG-LA^*, *rin^1–175^*, *rin^129–492^* and *rin^445–689^*, respectively, were cloned into the Gal4 DNA-binding domain vector pDEST 32 as well as into the Gal4 activation domain vector pDEST 22. Plasmids were transformed into yeast strain AH109 and plated on SD-Leu-Trp-Ade and SD-Leu-Trp-His (supplemented with 2 mM 3-AT), respectively.

## Supporting Information

Figure S1 Effective downregulation of *lig* during development. (A–B) Animals mutant for *lig^2^* (A) or *lig^3^* (B) in combination with *lig^PP1^* die as long, slender pupae. Scale bar represents 500 µm. (C) Statistical analysis of the size of seven ommatidia as described in Figure 1D: control (0.095±0.0022 and 0.09±0.0055) and *lig^1^* mutant (0.097±0.005; p = 0.3 and 0.09±0.0017; p = 0.5) eyes of flies raised on 25% yeast-containing food. (D–G) Scanning electron micrographs of adult control and *lig^1^* mutant eyes generated by eyFLP/FRT-mediated mitotic recombination from flies grown on 40% yeast food (D–E) or 40% yeast and 60% Casein-containing food (F–G). Scale bar represents 100 µm. (H) Statistical analysis as described in Figure 1D: control (749±19 and 721±24) and *lig^1^* mutant (791±38; p = 0.025 and 765±35; p = 0.021) eyes at 40% yeast-containing food, and the control (751±14 and 706±26) and *lig* (761±40; p = 0.51 and 741±19; p = 0.016) mutant eyes at 40% yeast and 60% Casein-containing food. (I–J) Scanning electron micrographs of eyFLP/FRT Minute-induced adult control or *lig^1^* mutant eyes (I–J) from flies grown on 100% yeast-containing food. Scale bar represents 100 µm. (K) Statistical analysis as described in Figure 1D: control (806±15 and 781±14) and *lig^1^* (837±30; p = 0.028 and 844±45; p = 0.0091). (L) Statistical analysis as described in Figure 1D: control (771±19 and 753±23) and *lig^1^* mutant (713±60; p = 0.042 and 755±38; p = 0.91) eyes from flies raised on 100% yeast-containing food at 18°C. (M) Statistical analysis as described in Figure 1D: control (653±63) and *lig^1^* mutant (750±32; p = 0.003) eyes from flies raised on 25% yeast-containing food at 18°C. (N–P) Overexpression of the transgenes *UAS-lig^RNAi I^* (O) or *UAS-lig^RNAi II^* (P) under the control of *da-Gal4* causes lethality. Control flies are shown in (N). Scale bar represents 500 µm. (Q–S') Compartment-specific expression of the transgenes *UAS-lig^RNAi I^* (R) or *UAS-lig^RNAi II^* (S) driven by *DE-Gal4* in the developing eye results in reduction of Lig (green) in the dorsal compartment (marked with RFP (red)) in comparison to the control (Q). Scale bar represents 50 µm. (T–V) Eyes overexpressing the transgenes *UAS-lig^RNAi I^* (U) or *UAS-lig^RNAi II^* (V) are larger than the control (T). Scale bar represents 100 µm. The expression of the transgenes was induced with the Actin-Flp out technique in combination with *eyFLP*. (W) Statistical analysis as described in Figure 1D: control (764±14 and 780±20), *UAS-lig^RNAi I^* (784±25; p = 0.035 and 799±21; p = 0.101) and *UAS-lig^RNAi II^* (809±7; p = 3.95E-08 and 800±16; p = 0.061). (X) Statistical analysis of the size of seven ommatidia as described in Figure 1D: control (0.093±0.0021 and 0.092±0.0049), *UAS-lig^RNAi I^* (0.094±0.004; p = 0.58 and 0.093±0.008; p = 0.78) and *UAS-lig^RNAi II^* (0.095±0.0035; p = 0.17 and 0.096±0.0069; p = 0.25) expressing eyes. Genotypes: (A) *y w*; *lig^PP1^*/*FRT42 lig^2^* (B) *y w*; *lig^PP1^*/*FRT42 lig^3^* (D, F, L (control) and M (control)) *y w eyFLP*/*y w*; *FRT42 P{SUPor-P}VhaAC45^KG02272^* (cl)/*FRT42* (E, G, L (*lig^1^*) and M (*lig^1^*)) *y w eyFLP*/*y w*; *FRT42 P{SUPor-P}VhaAC45^KG02272^* (cl)/*FRT42 lig^1^* (I) *y w eyFLP*/*y w*; *FRT42 M(2)53^1^*/*FRT42* (J) *y w eyFLP*/*y w*; *FRT42 M(2)53^1^*/*FRT42 lig^1^* (N) *y w*; *da-Gal4*/*UAS-CG1315^RNAi^* (control) (O) *y w*; *da-Gal4*/*UAS-lig^RNAi I^* [86Fb] (P) *y w*; *da-Gal4*/*UAS-lig^RNAi II^* [86Fb] (Q) *y w*; *DE-Gal4*, *UAS-RFP*/*UAS-CG1315^RNAi^* (control) (R) *y w*; *DE-Gal4*, *UAS-RFP*/*UAS-lig^RNAi I^* [86Fb] (S) *y w*; *DE-Gal4*, *UAS-RFP*/*UAS-lig^RNAi II^* [86Fb] (T) *y w eyFLP*, *Act>CD2>Gal4*/*y w*; *UAS-CG1315^RNAi^* (control)/+ (U) *y w eyFLP*, *Act>CD2>Gal4*/*y w*; *UAS-lig^RNAi I^* [86Fb]/+ (V) *y w eyFLP*, *Act>CD2>Gal4*/*y w*; *UAS-lig^RNAi II^* [86Fb]/+.
(TIF)

Figure S2 Overexpression of *lig^R185C^* causes a similar eye phenotype as overexpression of *lig*. (A–B) Scanning electron micrographs of eyes overexpressing the indicated UAS transgenes (A–B). Scale bar represents 100 µm. (C) Statistical analyses as described in Figure 1D: *ey>GFP* (790±17 and 770±15), *ey>lig^R185C^* (708±30; p = 1.43E-04 and 707±15; p = 6.00E-06). The phenotype caused by *lig^R185C^* is very similar to the phenotype caused by *lig* (Figure 2E). (D–E') *lig* overexpressing clones (induced with the Actin-Flp out-Gal4 system and marked by GFP) in eye imaginal discs of third instar larvae undergo apoptosis as judged by Cleaved Caspase-3 staining (red) (E–E') in comparison to the control (D–D'). Scale bar represents 50 µm. (F–G) Pictures of wings expressing the indicated UAS transgenes under the control of *nubbin-Gal4* (F and G). Scale bar represents 100 µm. (H) Statistical analysis as described in Figure 1D: *nubbin>GFP* (276838±12458 and 274887±13574), *nubbin>Lig* (98346±8035; p = 3.1E-13 and 97511±9593; p = 7.33E-12). Genotypes: (A) *w*/*y w*; *ey-Gal4*/*UAS-GFP* (B) *w*/*y w*; *ey-Gal4*/+; *UAS-lig^R185C^*/+ (D) *y w hsFLP*/*y w*; *UAS-GFP*/+; *Act>CD2>Gal4*, *UAS-GFP*/+ (E) *y w hsFLP*/*y w*; *Act>CD2>Gal4*, *UAS-GFP*/*UAS-lig* [86Fb] (F) *y w*/Y; *nubbin-Gal4*/*UAS-GFP* (G) *y w*/Y; *nubbin-Gal4*/+; *UAS-lig*/+.
(TIF)

Figure S3 Lig does not co-localize with DART1, and endogenous Lig, FMR1 and Capr co-localize with Rin-Cherry. (A–E''') S2 cells co-transfected with *GFP-lig* (A, A'), *RFP-FMR1* (B, B'), *RFP-rin* (C, C'), *RFP-DART1* (D, D'), and *GFP-FMR1* (E, E'''), *RFP-rin* (E', E''') and *HA-lig^R185C^* (E'', E'''). S2 cells stained with DAPI (blue) to visualize DNA and with α-HA to visualize HA-Lig (E'', E'''). (F–F''') S2 cells co-transfected with *GFP-lig^R185C^* (F', F''') and *RFP-DART1* (F'', F''') do not reveal any co-localization. S2 cells were stained with DAPI (blue) to visualize DNA. Scale bar represents 25 µm. (G–G') Untransfected S2 cells stained for endogenous Ago1. S2 cells were stained with DAPI (blue) to visualize DNA. Scale bar represents 25 µm. (H–K''') S2 cells transiently transfected with *Grin^Cherry^* to express Rin-Cherry at endogenous levels. In most of the cells Rin-Cherry is homogeneously in the cytoplasm of transfected cells (H and H'). In few cells Rin-Cherry forms punctae (I'', J'' and K'') and localizes with Lig (I' and I'''), FMR1 (J' and J''') and Capr (K' and K'''). S2 cells were stained with DAPI (blue) to visualize DNA. Scale bar represents 25 µm. (L–L''') Untransfected S2 cells stained for endogenous Lig (L' and L''') and Capr (L'' and L'''). Lig and Capr localize in bigger punctae but not in cells with small punctae. S2 cells were stained with DAPI (blue) to visualize DNA. Scale bar represents 25 µm.
(TIF)

Figure S4 Lig and Rin fragments display no autoactivity in Y2H experiments. (A) Negative controls for Y2H interactions between Lig, Lig^FG-LA^, Rin, Rin^1–175^, Rin^129–492^ and the empty vector. Lig, Rin, Rin^1–175^, Rin^129–492^ and Lig^FG-LA^ fused to the AD and to the DBD, respectively, do not show autoactivity.
(TIF)

Figure S5 Analysis of *rin* hypomorphic alleles and the genomic rescue transgene *Grin^Cherry^*. (A–C'') Negatively marked 72 h old *rin^2^* (A, A''), *P{GawB}rin^NP3248^* (B, B'') and *P{GawB}rin^NP5420^* (C, C'') mutant clones (induced with the FLP/FRT system) in eye imaginal discs of third instar larvae. Rin-Cherry levels expressed from the *Grin^Cherry^* are autonomously increased in the *rin* mutant clones (A', B' and C'). The scale bar represents 50 µm. (D–F) Scanning electron micrographs of adult *P{GawB}rin^NP3248^* (E) and *P{GawB}rin^NP5420^* (F) eyes generated by eyFLP/FRT-mediated mitotic recombination. The scale bar represents 100 µm. (G) Statistical analyses as described in Figure 1D: control (762±35 and 767±18), *P{GawB}rin^NP3248^* (732±9 and 731±17) and *P{GawB}rin^NP5420^* (713±20 and 711±29). (H–S) The long slender pupae formed by *FMR1^D113M/D50M^ rin^NP3248/2^* (K) and *FMR1^D113M/D50M^ rin^NP5420/2^* (Q) are rescued with one copy of the *Grin^Cherry^* transgene (M, S). The controls (heterozygous for the *FMR1* or *rin* alleles, respectively) do not show any defects (H–J, L, N–P and R). (T) Statistical analysis of the rescue of *FMR1^D113M^ rin^2^* mutant eyes with *Grin* as described in Figure 1D: control (765±13 and 744±14), *FMR1^D113M^ rin^2^* (800±27; p = 0.016 and not determined (ND)), *Grin*; *FMR1^D113M^ rin^2^* (754±20; p = 0.25 and 748±23; p = 0.7). Genotypes: (A) *y w hsFLP*/*y w*; *Grin^Cherry^* [44F]/+; *FRT82 ubiGFP*/*FRT82 rin^2^* (B) *y w hsFLP*/*y w*; *Grin^Cherry^* [44F]/+; *FRT82 ubiGFP*/*FRT82 P{GawB}rin^NP3248^* (C) *y w hsFLP*/*y w*; *Grin^Cherry^* [44F]/+; *FRT82 ubiGFP*/*FRT82 P{GawB}rin^NP5420^* (D) *y w eyFLP*/*y w*; *FRT82 cl w^+^*/*FRT82* (E) *y w eyFLP*/*y w*; *FRT82 cl w^+^*/*FRT82 P{GawB}rin^NP3248^* (F) *y w eyFLP*/*y w*; *FRT82 cl w^+^*/*FRT82 P{GawB}rin^NP5420^* (H) *y w*; *FRT82*/*FRT82 FMR1^D50M^ P{GawB}rin^NP3248^* (I) *y w*; *FRT82 FMR1^D113M^*/*FRT82 P{GawB}rin^NP3248^* (J) *y w*; *FRT82 FMR1^D113M^ rin^2^*/*FRT82 P{GawB}rin^NP3248^* (K) *y w eyFLP*/*y w*; *FRT82 FMR1^D113M^ rin^2^*/*FRT82 FMR1^D50M^ P{GawB}rin^NP3248^* (L) *y w eyFLP*/*y w*; *Grin^Cherry^* [44F]/+; *FRT82*/*FRT82 FMR1^D50M^ P{GawB}rin^NP3248^* (M) *y w eyFLP*/*y w*; *Grin^Cherry^* [44F]/+; *FRT82 FMR1^D113M^ rin^2^*/*FRT82 FMR1^D50M^ P{GawB}rin^NP3248^* (N) *y w*; *FRT82*/*FRT82 FMR1^D50M^ P{GawB}rin^NP5420^* (O) *y w*/*y w*; *FRT82 FMR1^D113M^*/*FRT82 P{GawB}rin^NP5420^* (P) *y w*/*y w*; *FRT82 rin^2^*/*FRT82 FMR1^D50M^ P{GawB}rin^NP5420^* (Q) *y w eyFLP*/*y w*; *FRT82 FMR1^D113M^ rin^2^*/*FRT82 FMR1^D50M^ P{GawB}rin^NP5420^* (R) *y w*; *Grin^Cherry^* [44F]/+; *FRT82*/*FRT82 FMR1^D50M^ P{GawB}rin^NP5420^* (S) *y w eyFLP*/*y w*; *Grin^Cherry^* [44F]/+; *FRT82 FMR1^D113M^ rin^2^*/*FRT82 FMR1^D50M^ P{GawB}rin^NP5420^* (T – control) *y w eyFLP*/*y w*; *FRT82 cl w^+^*/*FRT82* (T - *FMR1^D113M^ rin^2^*) *y w eyFLP*/*y w*; *FRT82 cl w^+^*/*FRT82 FMR1^D113M^ rin^2^* (T - *Grin*; *FMR1^D113M^ rin^2^*) *y w eyFLP*/*y w*; *Grin* [44F]/+; *FRT82 cl w^+^*/*FRT82 FMR1^D113M^ rin^2^*.
(TIF)

Figure S6 *Capr^RNAi^* strongly reduces Capr levels. (A–A'') *Capr^RNAi^* overexpressing clones (induced with the Actin-Flp out-Gal4 system and marked by GFP) in eye imaginal discs of third instar larvae reduce Capr levels as judged by Capr staining (red) (A'–A''). Scale bar represents 50 µm. Genotypes: (A) *y w hsFLP*/*y w*; *UAS-Capr^RNAi^*/+; *Act>CD2>Gal4*, *UAS-GFP*/+.
(TIF)

Figure S7 Lig regulates Rin levels but not at the protein level. (A–A'') *lig* overexpressing clones (induced with the Actin-Flp out system and marked by GFP) (A and A'') display increased levels of Rin-Cherry expressed from the *Grin^Cherry^* transgene (red) (A' and A''). Scale bar represents 50 µm. (B–B'') Negatively marked 72 h old *lig^1^* mutant clones (induced with the FLP/FRT system) in eye imaginal discs of third instar larvae expressing *UAS-HA-rin* under the control of *ey-* and *GMR-Gal4* (B' and B''). Note that HA-Rin is more strongly expressed in the posterior part of the disc due to the strong expression of Gal4 by *GMR-Gal4*. Scale bar represents 50 µm. Genotypes: (A) *y w hsFLP*/*y w*; *Grin^Cherry^* [44F]/+; *Act>CD2>Gal4*, *UAS-GFP*/*UAS-lig* [86Fb] (B) *y w hsFLP*/*y w*; *FRT42 ubiGFP*/*FRT42 lig^1^*; *ey-Gal4*, *GMR-Gal4*/*UAS-HA-rin* [86Fb].
(TIF)

Figure S8 Lig does not regulate *bantam* miRNA, EGFR signaling, Myc, Hippo signaling, Insulin signaling, Wnt signaling and Hedgehog signaling. (A–A'') *lig^1^* mutant clones (induced with the FLP/FRT system, 72 h old, marked by the lack of lacZ staining in red) in eye imaginal discs of third instar larvae (A and A'') do not display an upregulation of a minimal Hippo response element (DIAP1-GFP4.3; green; A' and A''). Scale bar represents 50 µm. (B–C'') Negatively marked 72 h old *lig^3^* mutant clones (induced with the FLP/FRT system; no lacZ (red); B and B'') and *lig* overexpressing cells (induced with the Gal4/UAS system using *DE-Gal4*) marked with RFP (C and C'') in eye imaginal discs of third instar larvae do not impact on a *bantam* miRNA reporter (B', B'', C' and C''). Scale bar represents 50 µm. (D–K'') Negatively marked 72 h old *lig^1^* mutant clones (induced with the FLP/FRT system; no GFP (green)) in wing (G, G'', H, H'', J, J'', K and K'') or eye (D, D'', E, E'', F, F'', I and I'') imaginal discs do not change expression or localization of *pnt-lacZ* (D' and D''), pAkt (E' and E''), Myc (F' and F''), Dll (G' and G''), Sens (H', H'', I' and I''), Ptc (J' and J'') and Cut (K' and K''). Scale bars represent 50 µm. (L–L'') Negatively marked 72 h old *lig1* mutant clones (induced with the FLP/FRT system; no GFP (green)) in eye imaginal discs stained for STAT92E (L' and L''). Scale bars represent 50 µm. Genotypes: (A) *y w hsFLP*/*y w*; *FRT42 arm-lacZ*/*FRT42 lig^1^*; *DIAP1-GFP4.3*/+ (B) *y w hsFLP*/*y w*; *FRT42 arm-lacZ*/*FRT42 lig^3^*; *MIR33 bantam reporter*/+ (C) *y w*/*y w*; *UAS-lig*/*+*; *MIR33 bantam reporter*/*DE-Gal4*, *UAS-RFP* (D) *y w hsFLP*/*y w*; *FRT42 ubiGFP*/*FRT42 lig^1^*; *pnt-lacZ*/+ (E, F, G, H, I, J, K, L) *y w hsFLP*/*y w*; *FRT42 ubiGFP*/*FRT42 lig^1^*.
(TIF)

Table S1 Lig interaction partners identified in AP-MS experiments. HA-GFP and HA-Lig expressed under the control of a metallothionein-inducible promoter in cultured *Drosophila* S2 cells were used as bait for AP-MS analyses. The unique and total peptide numbers identified in two biological replicates are indicated for HA-GFP (control) and HA-Lig. FlyBase ID and gene symbols of the corresponding genes are listed.
(XLSX)

## References

[1] Tumaneng K, Russell RC, Guan K-L (2012) Organ size control by Hippo and TOR pathways. Curr Biol 22: R368–R379. 22575479 10.1016/j.cub.2012.03.003 PMC3601681

[2] Luo Y, Shan G, Guo W, Smrt RD, Johnson EB, et al (2010) Fragile X mental retardation protein regulates proliferation and differentiation of adult neural stem/progenitor cells. PLoS Genet 6: e1000898. 20386739 10.1371/journal.pgen.1000898 PMC2851565

[3] Lypowy J, Chen I-Y, Abdellatif M (2005) An alliance between Ras GTPase-activating protein, filamin C, and Ras GTPase-activating protein SH3 domain-binding protein regulates myocyte growth. J Biol Chem 280: 25717–25728. 15886195 10.1074/jbc.M414266200

[4] Solomon S, Xu Y, Wang B, David MD, Schubert P, et al (2007) Distinct structural features of caprin-1 mediate its interaction with G3BP-1 and its induction of phosphorylation of eukaryotic translation initiation factor 2alpha, entry to cytoplasmic stress granules, and selective interaction with a subset of mRNAs. Mol Cell Biol 27: 2324–2342. 17210633 10.1128/MCB.02300-06 PMC1820512

[5] Bikkavilli RK, Malbon CC (2011) Arginine methylation of G3BP1 in response to Wnt3a regulates β-catenin mRNA. J Cell Sci 124: 2310–2320. 21652632 10.1242/jcs.084046 PMC3113675

[6] Yang Y, Xu S, Xia L, Wang J, Wen S, et al (2009) The *bantam* microRNA is associated with *Drosophila* fragile X mental retardation protein and regulates the fate of germline stem cells. PLoS Genet 5: e1000444. 19343200 10.1371/journal.pgen.1000444 PMC2654963

[7] Callan MA, Cabernard C, Heck J, Luois S, Doe CQ, et al (2010) Fragile X protein controls neural stem cell proliferation in the *Drosophila* brain. Hum Mol Genet 19: 3068–3079. 20504994 10.1093/hmg/ddq213 PMC2901145

[8] Fatimy El R, Tremblay S, Dury AY, Solomon S, De Koninck P, et al (2012) Fragile X mental retardation protein interacts with the RNA-binding protein Caprin1 in neuronal RiboNucleoProtein complexes. PLoS ONE 7: e39338. 22737234 10.1371/journal.pone.0039338 PMC3380850

[9] Papoulas O, Monzo KF, Cantin GT, Ruse C, Yates JR, et al (2010) dFMRP and Caprin, translational regulators of synaptic plasticity, control the cell cycle at the *Drosophila* mid-blastula transition. Development 137: 4201–4209. 21068064 10.1242/dev.055046 PMC2990211

[10] Grill B, Wilson GM, Zhang K-X, Wang B, Doyonnas R, et al (2004) Activation/division of lymphocytes results in increased levels of cytoplasmic activation/proliferation-associated protein-1: prototype of a new family of proteins. J Immunol 172: 2389–2400. 14764709 10.4049/jimmunol.172.4.2389

[11] Wang B, David MD, Schrader JW (2005) Absence of caprin-1 results in defects in cellular proliferation. J Immunol 175: 4274–4282. 16177067 10.4049/jimmunol.175.7.4274

[12] Gallouzi IE, Parker F, Chebli K, Maurier F, Labourier E, et al (1998) A novel phosphorylation-dependent RNase activity of GAP-SH3 binding protein: a potential link between signal transduction and RNA stability. Mol Cell Biol 18: 3956–3965. 9632780 10.1128/mcb.18.7.3956 PMC108980

[13] Tourrière H, Gallouzi IE, Chebli K, Capony JP, Mouaikel J, et al (2001) RasGAP-associated endoribonuclease G3Bp: selective RNA degradation and phosphorylation-dependent localization. Mol Cell Biol 21: 7747–7760. 11604510 10.1128/MCB.21.22.7747-7760.2001 PMC99945

[14] Hafen E (2004) Cancer, type 2 diabetes, and ageing: news from flies and worms. Swiss Med Wkly 134: 711–719. 15635489 10.4414/smw.2004.09885

[15] Kuniyoshi H, Baba K, Ueda R, Kondo S, Awano W, et al (2002) *lingerer*, a *Drosophila* gene involved in initiation and termination of copulation, encodes a set of novel cytoplasmic proteins. Genetics 162: 1775–1789. 12524348 10.1093/genetics/162.4.1775 PMC1462391

[16] Ryoo HD, Bergmann A, Gonen H, Ciechanover A, Steller H (2002) Regulation of *Drosophila* IAP1 degradation and apoptosis by reaper and ubcD1. Nat Cell Biol 4: 432–438. 12021769 10.1038/ncb795

[17] Martin FA, Pérez-Garijo A, Morata G (2009) Apoptosis in *Drosophila*: compensatory proliferation and undead cells. Int J Dev Biol 53: 1341–1347. 19247932 10.1387/ijdb.072447fm

[18] Costa A, Wang Y, Dockendorff TC, Erdjument-Bromage H, Tempst P, et al (2005) The *Drosophila* fragile X protein functions as a negative regulator in the orb autoregulatory pathway. Dev Cell 8: 331–342. 15737929 10.1016/j.devcel.2005.01.011

[19] Barbee SA, Estes PS, Cziko A-M, Hillebrand J, Luedeman RA, et al (2006) Staufen- and FMRP-containing neuronal RNPs are structurally and functionally related to somatic P bodies. Neuron 52: 997–1009. 17178403 10.1016/j.neuron.2006.10.028 PMC1955741

[20] Beerman RW, Jongens TA (2011) A non-canonical start codon in the *Drosophila* fragile X gene yields two functional isoforms. Neuroscience 181: 48–66. 21333716 10.1016/j.neuroscience.2011.02.029 PMC3074047

[21] Behm-Ansmant I, Rehwinkel J, Doerks T, Stark A, Bork P, et al (2006) mRNA degradation by miRNAs and GW182 requires both CCR4∶NOT deadenylase and DCP1∶DCP2 decapping complexes. Genes Dev 20: 1885–1898. 16815998 10.1101/gad.1424106 PMC1522082

[22] Stewart M, Baker RP, Bayliss R, Clayton L, Grant RP, et al (2001) Molecular mechanism of translocation through nuclear pore complexes during nuclear protein import. FEBS Lett 498: 145–149. 11412846 10.1016/s0014-5793(01)02489-9

[23] Fribourg S, Braun IC, Izaurralde E, Conti E (2001) Structural basis for the recognition of a nucleoporin FG repeat by the NTF2-like domain of the TAP/p15 mRNA nuclear export factor. Mol Cell 8: 645–656. 11583626 10.1016/s1097-2765(01)00348-3

[24] Bayliss R, Littlewood T, Strawn LA, Wente SR, Stewart M (2002) GLFG and FxFG nucleoporins bind to overlapping sites on importin-beta. J Biol Chem 277: 50597–50606. 12372823 10.1074/jbc.M209037200

[25] Vognsen T, Kristensen O (2012) Crystal structure of the Rasputin NTF2-like domain from *Drosophila melanogaster* . Biochem Biophys Res Commun 420: 188–192. 22414690 10.1016/j.bbrc.2012.02.140

[26] Franco M, Seyfried NT, Brand AH, Peng J, Mayor U (2011) A novel strategy to isolate ubiquitin conjugates reveals wide role for ubiquitination during neural development. Mol Cell Proteomics 10: M110.002188. 20861518 10.1074/mcp.M110.002188 PMC3098581

[27] Nolo R, Morrison CM, Tao C, Zhang X, Halder G (2006) The *bantam* microRNA is a target of the Hippo tumor-suppressor pathway. Curr Biol 16: 1895–1904. 16949821 10.1016/j.cub.2006.08.057

[28] Thompson BJ, Cohen SM (2006) The Hippo pathway regulates the *bantam* microRNA to control cell proliferation and apoptosis in *Drosophila* . Cell 126: 767–774. 16923395 10.1016/j.cell.2006.07.013

[29] Brennecke J, Hipfner DR, Stark A, Russell RB, Cohen SM (2003) *bantam* encodes a developmentally regulated microRNA that controls cell proliferation and regulates the proapoptotic gene *hid* in *Drosophila* . Cell 113: 25–36. 12679032 10.1016/s0092-8674(03)00231-9

[30] Epstein AM, Bauer CR, Ho A, Bosco G, Zarnescu DC (2009) *Drosophila* Fragile X protein controls cellular proliferation by regulating cbl levels in the ovary. Dev Biol 330: 83–92. 19306863 10.1016/j.ydbio.2009.03.011

[31] Callan MA, Clements N, Ahrendt N, Zarnescu DC (2012) Fragile X protein is required for inhibition of insulin signaling and regulates glial-dependent neuroblast reactivation in the developing brain. Brain Res 1462: 151–161. 22513101 10.1016/j.brainres.2012.03.042

[32] Müller P, Kuttenkeuler D, Gesellchen V, Zeidler MP, Boutros M (2005) Identification of JAK/STAT signalling components by genome-wide RNA interference. Nature 436: 871–875. 16094372 10.1038/nature03869

[33] Kuniyoshi H, Usui-Aoki K, Juni N, Yamamoto D (2003) Expression analysis of the *lingerer* gene in the larval central nervous system of *Drosophila melanogaster* . J Neurogenet 17: 117–137. 14668197

[34] Naz RK, Dhandapani L (2010) Identification of human sperm proteins that interact with human zona pellucida 3 (ZP3) using yeast two-hybrid system. J Reprod Immunol 84: 24–31. 19945174 10.1016/j.jri.2009.10.006 PMC2819281

[35] Wilde IB, Brack M, Winget JM, Mayor T (2011) Proteomic characterization of aggregating proteins after the inhibition of the ubiquitin proteasome system. J Proteome Res 10: 1062–1072. 21204586 10.1021/pr1008543

[36] Cziko A-MJ, McCann CT, Howlett IC, Barbee SA, Duncan RP, et al (2009) Genetic modifiers of dFMR1 encode RNA granule components in *Drosophila* . Genetics 182: 1051–1060. 19487564 10.1534/genetics.109.103234 PMC2728847

[37] Arbouzova NI, Zeidler MP (2006) JAK/STAT signalling in *Drosophila*: insights into conserved regulatory and cellular functions. Development 133: 2605–2616. 16794031 10.1242/dev.02411

[38] Yoon WH, Meinhardt H, Montell DJ (2011) miRNA-mediated feedback inhibition of JAK/STAT morphogen signalling establishes a cell fate threshold. Nat Cell Biol 13: 1062–1069. 21857668 10.1038/ncb2316 PMC3167036

[39] Luo W, Sehgal A (2012) Regulation of circadian behavioral output via a microRNA-JAK/STAT circuit. Cell 148: 765–779. 22305007 10.1016/j.cell.2011.12.024 PMC3307393

[40] Atlas R, Behar L, Elliott E, Ginzburg I (2004) The insulin-like growth factor mRNA binding-protein IMP-1 and the Ras-regulatory protein G3BP associate with *tau* mRNA and HuD protein in differentiated P19 neuronal cells. J Neurochem 89: 613–626. 15086518 10.1111/j.1471-4159.2004.02371.x

[41] Atlas R, Behar L, Sapoznik S, Ginzburg I (2007) Dynamic association with polysomes during P19 neuronal differentiation and an untranslated-region-dependent translation regulation of the *tau* mRNA by the *tau* mRNA-associated proteins IMP1, HuD, and G3BP1. J Neurosci Res 85: 173–183. 17086542 10.1002/jnr.21099

[42] Zhang YQ, Bailey AM, Matthies HJ, Renden RB, Smith MA, et al (2001) *Drosophila* fragile X-related gene regulates the MAP1B homolog Futsch to control synaptic structure and function. Cell 107: 591–603. 11733059 10.1016/s0092-8674(01)00589-x

[43] Bettencourt da Cruz A, Schwärzel M, Schulze S, Niyyati M, Heisenberg M, et al (2005) Disruption of the MAP1B-related protein FUTSCH leads to changes in the neuronal cytoskeleton, axonal transport defects, and progressive neurodegeneration in *Drosophila* . Mol Biol Cell 16: 2433–2442. 15772149 10.1091/mbc.E04-11-1004 PMC1087247

[44] Gary JD, Clarke S (1998) RNA and protein interactions modulated by protein arginine methylation. Prog Nucleic Acid Res Mol Biol 61: 65–131. 9752719 10.1016/s0079-6603(08)60825-9

[45] Bedford MT, Clarke SG (2009) Protein arginine methylation in mammals: who, what, and why. Mol Cell 33: 1–13. 19150423 10.1016/j.molcel.2008.12.013 PMC3372459

[46] Stetler A, Winograd C, Sayegh J, Cheever A, Patton E, et al (2006) Identification and characterization of the methyl arginines in the fragile X mental retardation protein Fmrp. Hum Mol Genet 15: 87–96. 16319129 10.1093/hmg/ddi429

[47] Pahlich S, Zakaryan RP, Gehring H (2008) Identification of proteins interacting with Protein arginine methyltransferase 8: the Ewing sarcoma (EWS) protein binds independent of its methylation state. Proteins 72: 1125–1137. 18320585 10.1002/prot.22004

[48] Pazman C, Mayes CA, Fanto M, Haynes SR, Mlodzik M (2000) Rasputin, the *Drosophila* homologue of the RasGAP SH3 binding protein, functions in ras- and Rho-mediated signaling. Development 127: 1715–1725. 10725247 10.1242/dev.127.8.1715

[49] Newsome TP, Asling B, Dickson BJ (2000) Analysis of *Drosophila* photoreceptor axon guidance in eye-specific mosaics. Development 127: 851–860. 10648243 10.1242/dev.127.4.851

[50] Neufeld TP, la Cruz de AF, Johnston LA, Edgar BA (1998) Coordination of growth and cell division in the *Drosophila* wing. Cell 93: 1183–1193. 9657151 10.1016/s0092-8674(00)81462-2

[51] Calleja M, Moreno E, Pelaz S, Morata G (1996) Visualization of gene expression in living adult *Drosophila* . Science 274: 252–255. 8824191 10.1126/science.274.5285.252

[52] Morrison CM, Halder G (2009) Characterization of a dorsal-eye Gal4 Line in *Drosophila* . Genesis 48: 3–7. 19882738 10.1002/dvg.20571 PMC3162325

[53] Hazelett DJ, Bourouis M, Walldorf U, Treisman JE (1998) *decapentaplegic* and *wingless* are regulated by *eyes absent* and *eyegone* and interact to direct the pattern of retinal differentiation in the eye disc. Development 125: 3741–3751. 9716539 10.1242/dev.125.18.3741

[54] Knoblich JA, Sauer K, Jones L, Richardson H, Saint R, et al (1994) Cyclin E controls S phase progression and its down-regulation during *Drosophila* embryogenesis is required for the arrest of cell proliferation. Cell 77: 107–120. 8156587 10.1016/0092-8674(94)90239-9

[55] Dockendorff TC, Su HS, McBride SMJ, Yang Z, Choi CH, et al (2002) *Drosophila* lacking dfmr1 activity show defects in circadian output and fail to maintain courtship interest. Neuron 34: 973–984. 12086644 10.1016/s0896-6273(02)00724-9

[56] Zhang L, Ren F, Zhang Q, Chen Y, Wang B, et al (2008) The TEAD/TEF family of transcription factor Scalloped mediates Hippo signaling in organ size control. Dev Cell 14: 377–387. 18258485 10.1016/j.devcel.2008.01.006 PMC2292673

[57] Bach EA, Ekas LA, Ayala-Camargo A, Flaherty MS, Lee H, et al (2007) GFP reporters detect the activation of the *Drosophila* JAK/STAT pathway in vivo. Gene Expr Patterns 7: 323–331. 17008134 10.1016/j.modgep.2006.08.003

[58] Gluderer S, Oldham S, Rintelen F, Sulzer A, Schütt C, et al (2008) Bunched, the *Drosophila* homolog of the mammalian tumor suppressor TSC-22, promotes cellular growth. BMC Dev Biol 8: 10. 18226226 10.1186/1471-213X-8-10 PMC2253523

[59] Venken K, Carlson J, Schulze K, Pan H, He Y, et al (2009) Versatile P[acman] BAC libraries for transgenesis studies in *Drosophila melanogaster* . Nat Methods 6: 431–434. 19465919 10.1038/nmeth.1331 PMC2784134

[60] Bischof J, Maeda RK, Hediger M, Karch F, Basler K (2007) An optimized transgenesis system for *Drosophila* using germ-line-specific phiC31 integrases. Proc Natl Acad Sci USA 104: 3312–3317. 17360644 10.1073/pnas.0611511104 PMC1805588

[61] Eulalio A, Behm-Ansmant I, Schweizer D, Izaurralde E (2007) P-body formation is a consequence, not the cause, of RNA-mediated gene silencing. Mol Cell Biol 27: 3970–3981. 17403906 10.1128/MCB.00128-07 PMC1900022

[62] Gluderer S, Brunner E, Germann M, Jovaisaite V, Li C, et al (2010) Madm (Mlf1 adapter molecule) cooperates with Bunched A to promote growth in *Drosophila* . J Biol 9: 9. 20149264 10.1186/jbiol216 PMC2871527

[63] Miyoshi K, Tsukumo H, Nagami T, Siomi H, Siomi MC (2005) Slicer function of *Drosophila* Argonautes and its involvement in RISC formation. Genes Dev 19: 2837–2848. 16287716 10.1101/gad.1370605 PMC1315391

[64] Wan L, Dockendorff TC, Jongens TA, Dreyfuss G (2000) Characterization of dFMR1, a *Drosophila melanogaster* homolog of the fragile X mental retardation protein. Mol Cell Biol 20: 8536–8547. 11046149 10.1128/mcb.20.22.8536-8547.2000 PMC102159

[65] Maines JZ, Stevens LM, Tong X, Stein D (2004) *Drosophila* dMyc is required for ovary cell growth and endoreplication. Development 131: 775–786. 14724122 10.1242/dev.00932

[66] Chen H-W, Chen X, Oh S-W, Marinissen MJ, Gutkind JS, et al (2002) *mom* identifies a receptor for the *Drosophila* JAK/STAT signal transduction pathway and encodes a protein distantly related to the mammalian cytokine receptor family. Genes Dev 16: 388–398. 11825879 10.1101/gad.955202 PMC155335

