# Supplementary figures and images for: The RNA-binding Proteins FMR1, Rasputin and Caprin Act Together with the UBA Protein Lingerer to Restrict Tissue Growth in Drosophila melanogaster
Source: PLoS Genet. 2013 Jul 11;9(7):e1003598. doi: 10.1371/journal.pgen.1003598 (PMC3708825; doi:10.1371/journal.pgen.1003598)

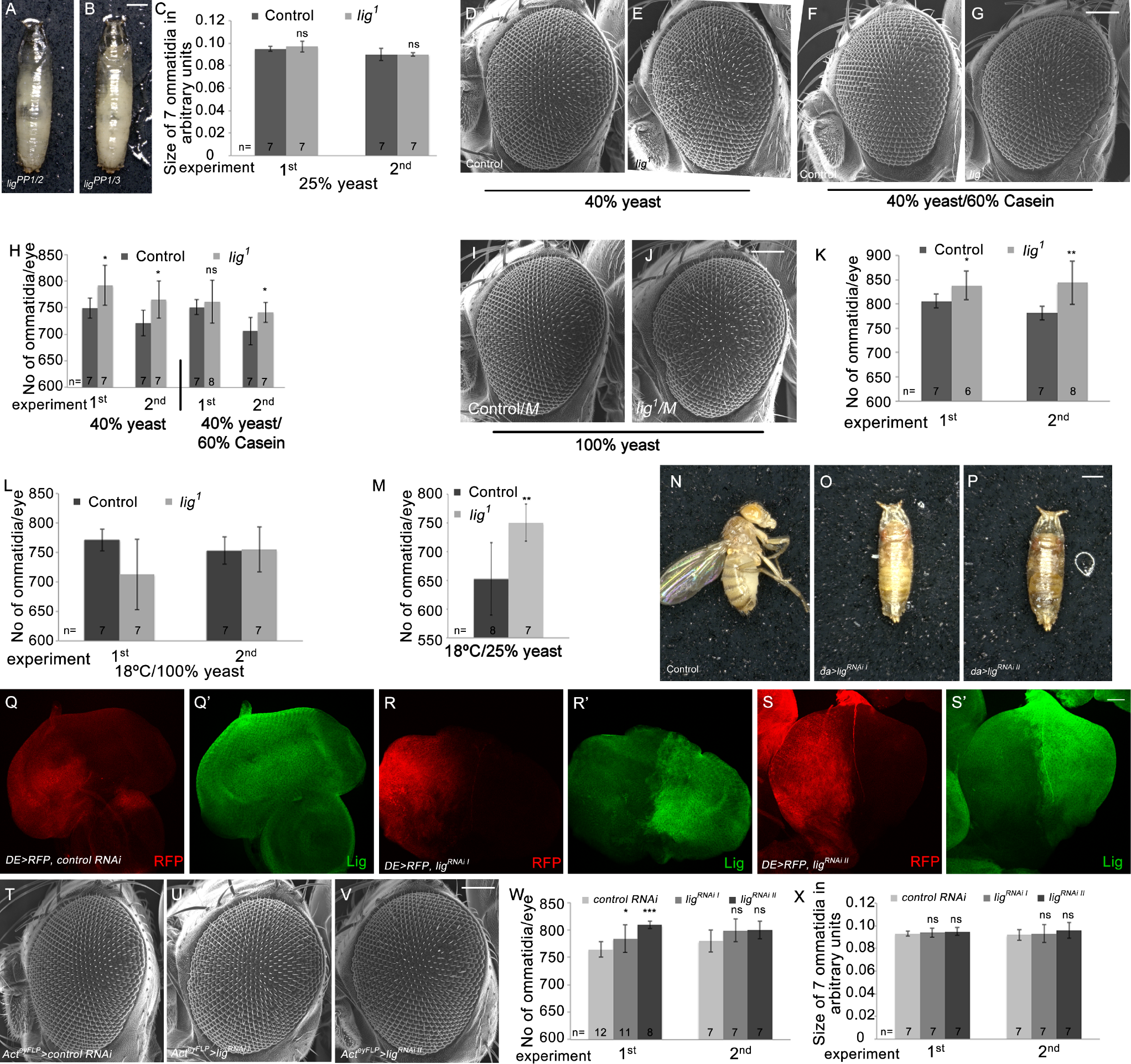

Supplement: Figure S1 — Effective downregulation of lig during development. (A–B) Animals mutant for lig2 (A) or lig3 (B) in combination with ligPP1 die as long, slender pupae. Scale bar represents 500 µm. (C) Statistical analysis of the size of seven ommatidia as described in Figure 1D: control (0.095±0.0022 and 0.09±0.0055) and lig1 mutant (0.097±0.005; p = 0.3 and 0.09±0.0017; p = 0.5) eyes of flies raised on 25% yeast-containing food. (D–G) Scanning electron micrographs of adult control and lig1 mutant eyes generated by eyFLP/FRT-mediated mitotic recombination from flies grown on 40% yeast food (D–E) or 40% yeast and 60% Casein-containing food (F–G). Scale bar represents 100 µm. (H) Statistical analysis as described in Figure 1D: control (749±19 and 721±24) and lig1 mutant (791±38; p = 0.025 and 765±35; p = 0.021) eyes at 40% yeast-containing food, and the control (751±14 and 706±26) and lig (761±40; p = 0.51 and 741±19; p = 0.016) mutant eyes at 40% yeast and 60% Casein-containing food. (I–J) Scanning electron micrographs of eyFLP/FRT Minute-induced adult control or lig1 mutant eyes (I–J) from flies grown on 100% yeast-containing food. Scale bar represents 100 µm. (K) Statistical analysis as described in Figure 1D: control (806±15 and 781±14) and lig1 (837±30; p = 0.028 and 844±45; p = 0.0091). (L) Statistical analysis as described in Figure 1D: control (771±19 and 753±23) and lig1 mutant (713±60; p = 0.042 and 755±38; p = 0.91) eyes from flies raised on 100% yeast-containing food at 18°C. (M) Statistical analysis as described in Figure 1D: control (653±63) and lig1 mutant (750±32; p = 0.003) eyes from flies raised on 25% yeast-containing food at 18°C. (N–P) Overexpression of the transgenes UAS-ligRNAi I (O) or UAS-ligRNAi II (P) under the control of da-Gal4 causes lethality. Control flies are shown in (N). Scale bar represents 500 µm. (Q–S') Compartment-specific expression of the transgenes UAS-ligRNAi I (R) or UAS-ligRNAi II (S) driven by DE-Gal4 in the developing eye results in red [file pgen.1003598.s001.tif]

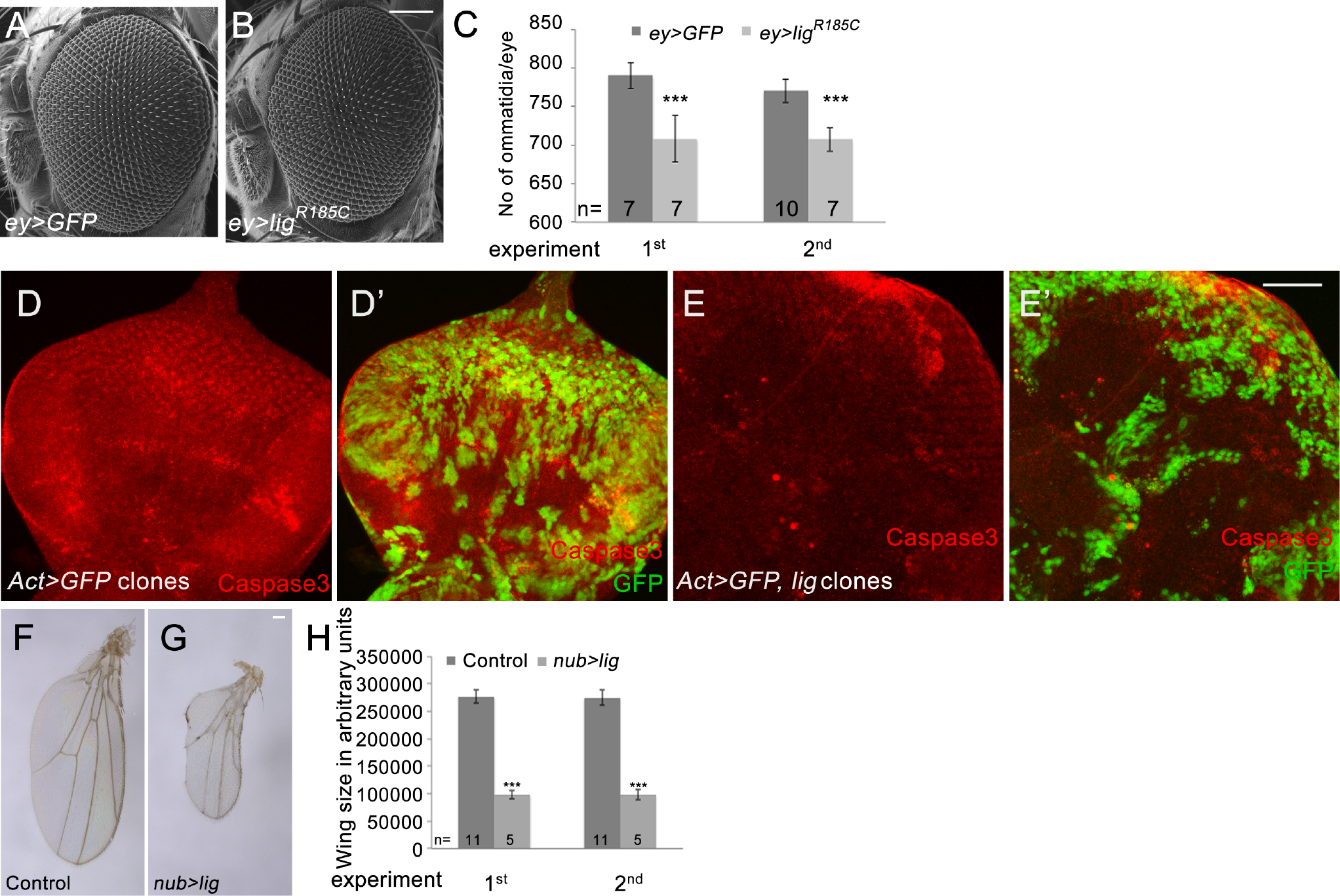

Supplement: Figure S2 — Overexpression of ligR185C causes a similar eye phenotype as overexpression of lig. (A–B) Scanning electron micrographs of eyes overexpressing the indicated UAS transgenes (A–B). Scale bar represents 100 µm. (C) Statistical analyses as described in Figure 1D: ey>GFP (790±17 and 770±15), ey>ligR185C (708±30; p = 1.43E-04 and 707±15; p = 6.00E-06). The phenotype caused by ligR185C is very similar to the phenotype caused by lig (Figure 2E). (D–E') lig overexpressing clones (induced with the Actin-Flp out-Gal4 system and marked by GFP) in eye imaginal discs of third instar larvae undergo apoptosis as judged by Cleaved Caspase-3 staining (red) (E–E') in comparison to the control (D–D'). Scale bar represents 50 µm. (F–G) Pictures of wings expressing the indicated UAS transgenes under the control of nubbin-Gal4 (F and G). Scale bar represents 100 µm. (H) Statistical analysis as described in Figure 1D: nubbin>GFP (276838±12458 and 274887±13574), nubbin>Lig (98346±8035; p = 3.1E-13 and 97511±9593; p = 7.33E-12). Genotypes: (A) w/y w; ey-Gal4/UAS-GFP (B) w/y w; ey-Gal4/+; UAS-ligR185C/+ (D) y w hsFLP/y w; UAS-GFP/+; Act>CD2>Gal4, UAS-GFP/+ (E) y w hsFLP/y w; Act>CD2>Gal4, UAS-GFP/UAS-lig [86Fb] (F) y w/Y; nubbin-Gal4/UAS-GFP (G) y w/Y; nubbin-Gal4/+; UAS-lig/+. (TIF) [file pgen.1003598.s002.tif]

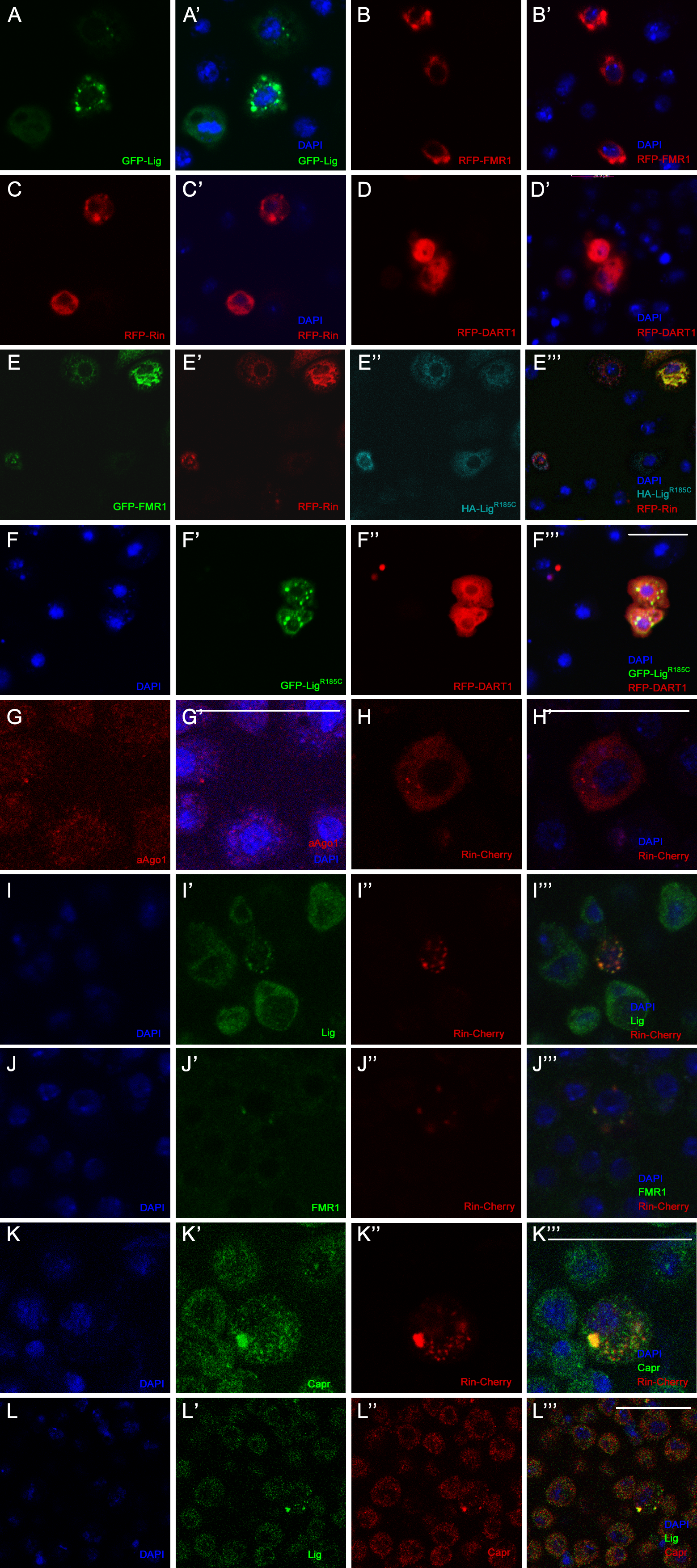

Supplement: Figure S3 — Lig does not co-localize with DART1, and endogenous Lig, FMR1 and Capr co-localize with Rin-Cherry. (A–E''') S2 cells co-transfected with GFP-lig (A, A'), RFP-FMR1 (B, B'), RFP-rin (C, C'), RFP-DART1 (D, D'), and GFP-FMR1 (E, E'''), RFP-rin (E', E''') and HA-ligR185C (E'', E'''). S2 cells stained with DAPI (blue) to visualize DNA and with α-HA to visualize HA-Lig (E'', E'''). (F–F''') S2 cells co-transfected with GFP-ligR185C (F', F''') and RFP-DART1 (F'', F''') do not reveal any co-localization. S2 cells were stained with DAPI (blue) to visualize DNA. Scale bar represents 25 µm. (G–G') Untransfected S2 cells stained for endogenous Ago1. S2 cells were stained with DAPI (blue) to visualize DNA. Scale bar represents 25 µm. (H–K''') S2 cells transiently transfected with GrinCherry to express Rin-Cherry at endogenous levels. In most of the cells Rin-Cherry is homogeneously in the cytoplasm of transfected cells (H and H'). In few cells Rin-Cherry forms punctae (I'', J'' and K'') and localizes with Lig (I' and I'''), FMR1 (J' and J''') and Capr (K' and K'''). S2 cells were stained with DAPI (blue) to visualize DNA. Scale bar represents 25 µm. (L–L''') Untransfected S2 cells stained for endogenous Lig (L' and L''') and Capr (L'' and L'''). Lig and Capr localize in bigger punctae but not in cells with small punctae. S2 cells were stained with DAPI (blue) to visualize DNA. Scale bar represents 25 µm. (TIF) [file pgen.1003598.s003.tif]

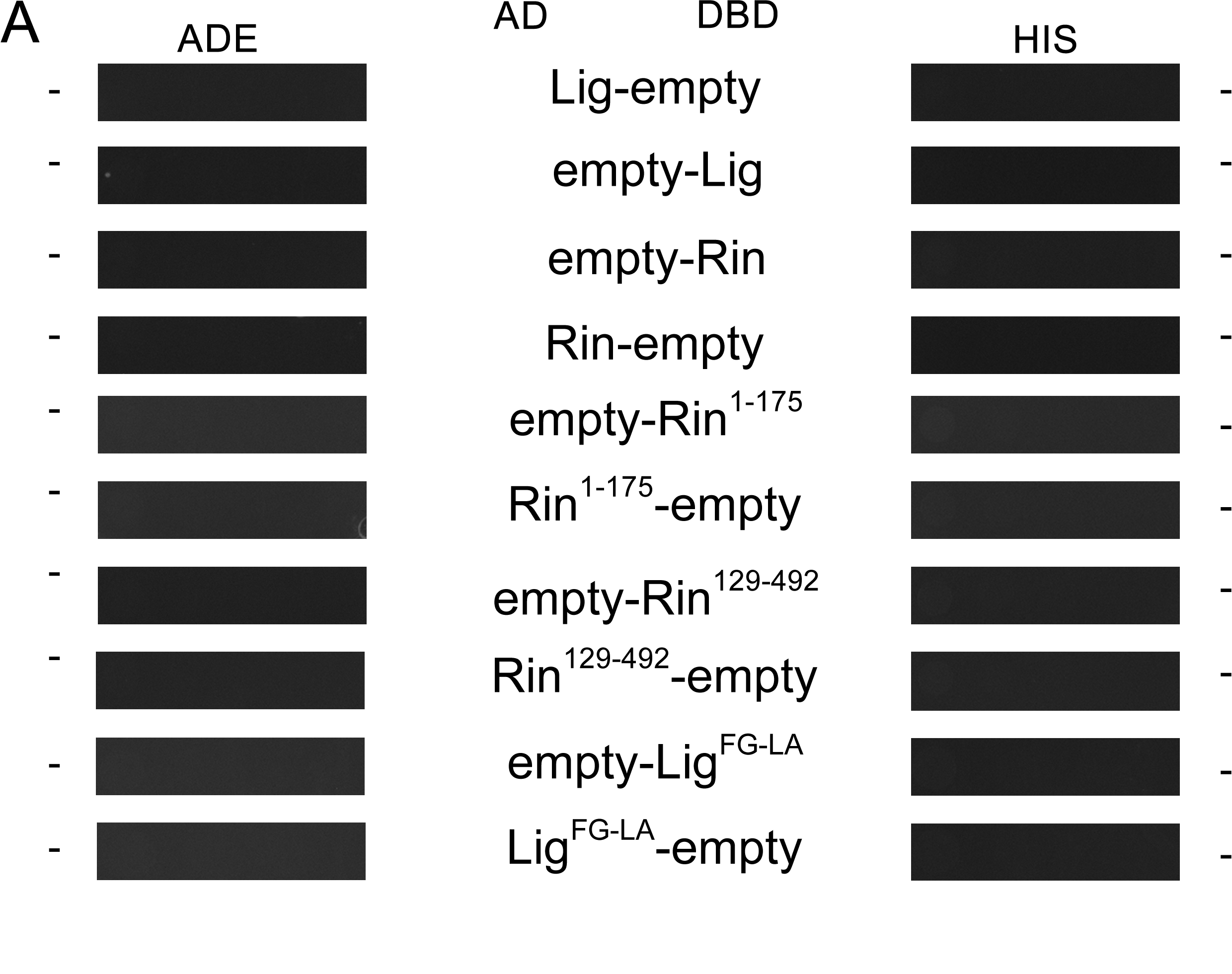

Supplement: Figure S4 — Lig and Rin fragments display no autoactivity in Y2H experiments. (A) Negative controls for Y2H interactions between Lig, LigFG-LA, Rin, Rin1–175, Rin129–492 and the empty vector. Lig, Rin, Rin1–175, Rin129–492 and LigFG-LA fused to the AD and to the DBD, respectively, do not show autoactivity. (TIF) [file pgen.1003598.s004.tif]

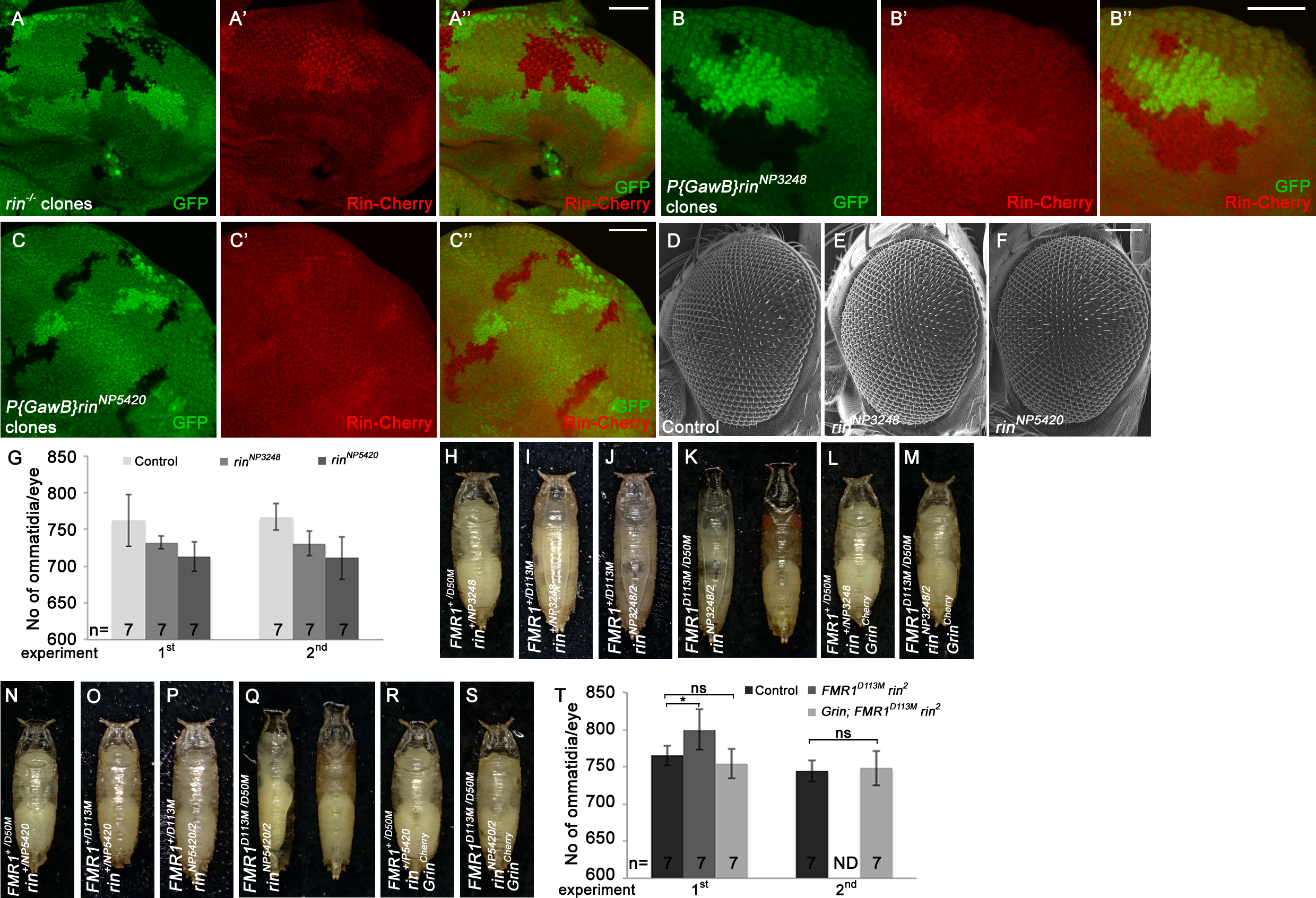

Supplement: Figure S5 — Analysis of rin hypomorphic alleles and the genomic rescue transgene GrinCherry. (A–C'') Negatively marked 72 h old rin2 (A, A''), P{GawB}rinNP3248 (B, B'') and P{GawB}rinNP5420 (C, C'') mutant clones (induced with the FLP/FRT system) in eye imaginal discs of third instar larvae. Rin-Cherry levels expressed from the GrinCherry are autonomously increased in the rin mutant clones (A', B' and C'). The scale bar represents 50 µm. (D–F) Scanning electron micrographs of adult P{GawB}rinNP3248 (E) and P{GawB}rinNP5420 (F) eyes generated by eyFLP/FRT-mediated mitotic recombination. The scale bar represents 100 µm. (G) Statistical analyses as described in Figure 1D: control (762±35 and 767±18), P{GawB}rinNP3248 (732±9 and 731±17) and P{GawB}rinNP5420 (713±20 and 711±29). (H–S) The long slender pupae formed by FMR1D113M/D50M rinNP3248/2 (K) and FMR1D113M/D50M rinNP5420/2 (Q) are rescued with one copy of the GrinCherry transgene (M, S). The controls (heterozygous for the FMR1 or rin alleles, respectively) do not show any defects (H–J, L, N–P and R). (T) Statistical analysis of the rescue of FMR1D113M rin2 mutant eyes with Grin as described in Figure 1D: control (765±13 and 744±14), FMR1D113M rin2 (800±27; p = 0.016 and not determined (ND)), Grin; FMR1D113M rin2 (754±20; p = 0.25 and 748±23; p = 0.7). Genotypes: (A) y w hsFLP/y w; GrinCherry [44F]/+; FRT82 ubiGFP/FRT82 rin2 (B) y w hsFLP/y w; GrinCherry [44F]/+; FRT82 ubiGFP/FRT82 P{GawB}rinNP3248 (C) y w hsFLP/y w; GrinCherry [44F]/+; FRT82 ubiGFP/FRT82 P{GawB}rinNP5420 (D) y w eyFLP/y w; FRT82 cl w+/FRT82 (E) y w eyFLP/y w; FRT82 cl w+/FRT82 P{GawB}rinNP3248 (F) y w eyFLP/y w; FRT82 cl w+/FRT82 P{GawB}rinNP5420 (H) y w; FRT82/FRT82 FMR1D50M P{GawB}rinNP3248 (I) y w; FRT82 FMR1D113M/FRT82 P{GawB}rinNP3248 (J) y w; FRT82 FMR1D113M rin2/FRT82 P{GawB}rinNP3248 (K) y w eyFLP/y w; FRT82 FMR1D113M rin2/FRT82 FMR1D50M P{GawB}rinNP3248 (L) y w eyFLP/y w; GrinCherry [44F]/+; FRT82/FRT82 FMR1D50M P{GawB}rinNP3248 (M) y w eyFLP/y w; Grin [file pgen.1003598.s005.tif]

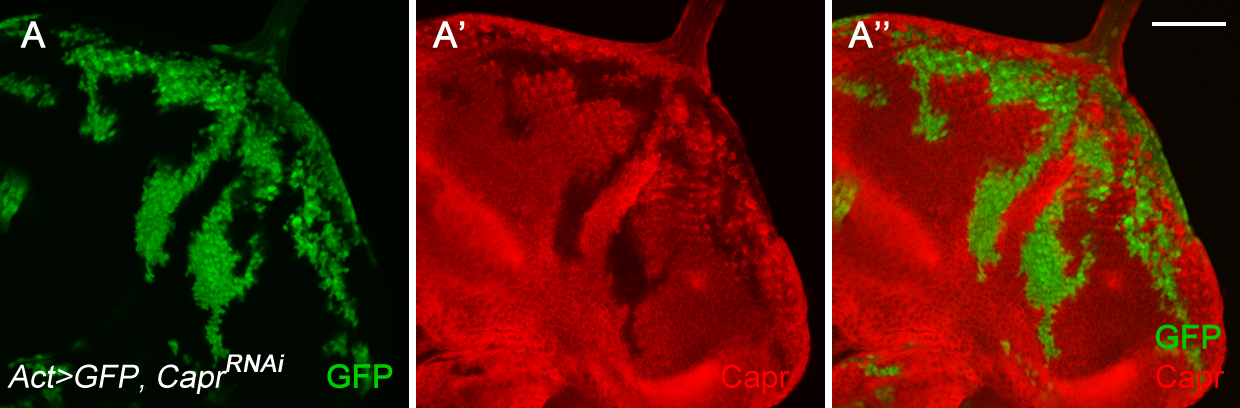

Supplement: Figure S6 — CaprRNAi strongly reduces Capr levels. (A–A'') CaprRNAi overexpressing clones (induced with the Actin-Flp out-Gal4 system and marked by GFP) in eye imaginal discs of third instar larvae reduce Capr levels as judged by Capr staining (red) (A'–A''). Scale bar represents 50 µm. Genotypes: (A) y w hsFLP/y w; UAS-CaprRNAi/+; Act>CD2>Gal4, UAS-GFP/+. (TIF) [file pgen.1003598.s006.tif]

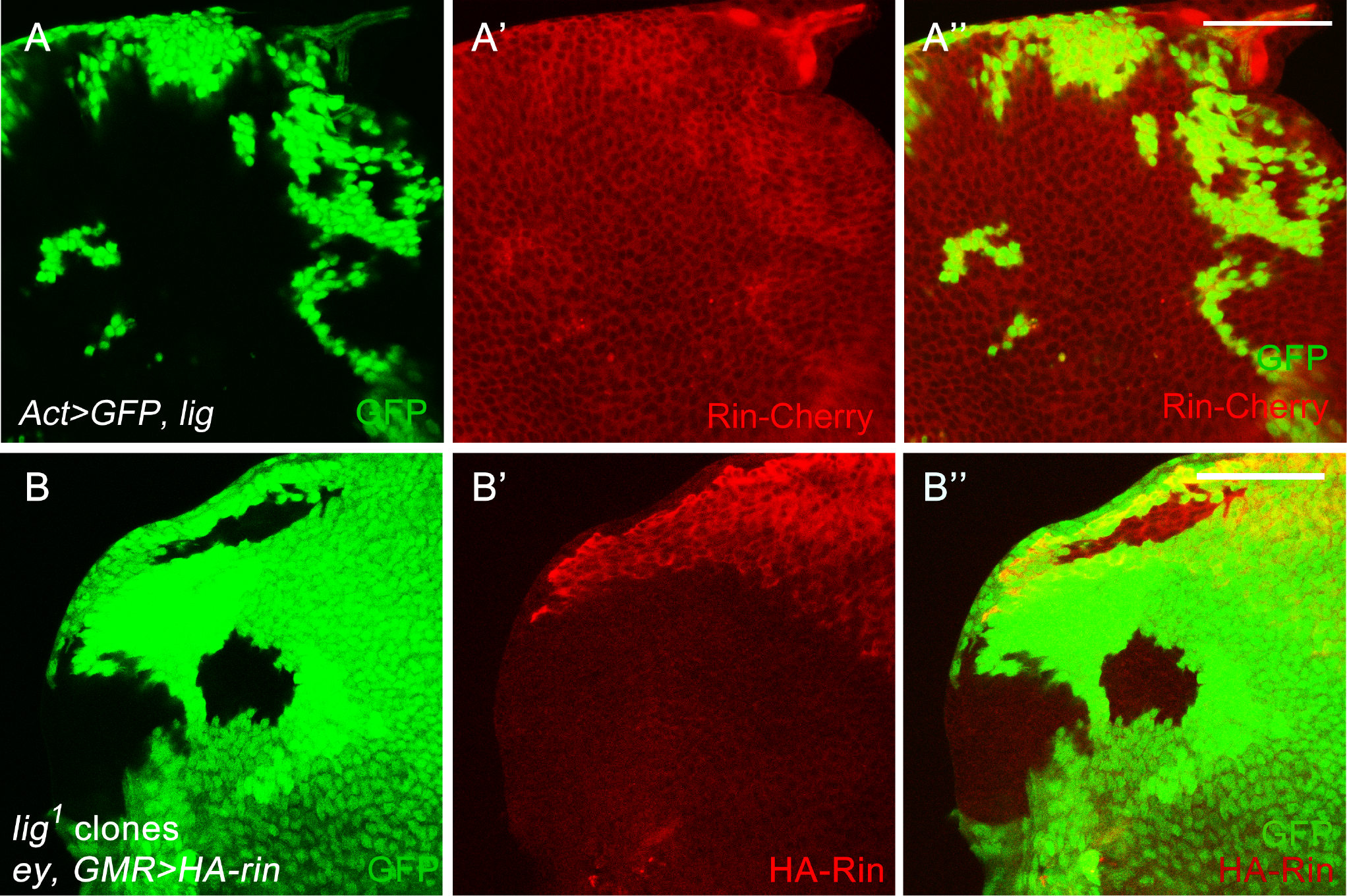

Supplement: Figure S7 — Lig regulates Rin levels but not at the protein level. (A–A'') lig overexpressing clones (induced with the Actin-Flp out system and marked by GFP) (A and A'') display increased levels of Rin-Cherry expressed from the GrinCherry transgene (red) (A' and A''). Scale bar represents 50 µm. (B–B'') Negatively marked 72 h old lig1 mutant clones (induced with the FLP/FRT system) in eye imaginal discs of third instar larvae expressing UAS-HA-rin under the control of ey- and GMR-Gal4 (B' and B''). Note that HA-Rin is more strongly expressed in the posterior part of the disc due to the strong expression of Gal4 by GMR-Gal4. Scale bar represents 50 µm. Genotypes: (A) y w hsFLP/y w; GrinCherry [44F]/+; Act>CD2>Gal4, UAS-GFP/UAS-lig [86Fb] (B) y w hsFLP/y w; FRT42 ubiGFP/FRT42 lig1; ey-Gal4, GMR-Gal4/UAS-HA-rin [86Fb]. (TIF) [file pgen.1003598.s007.tif]

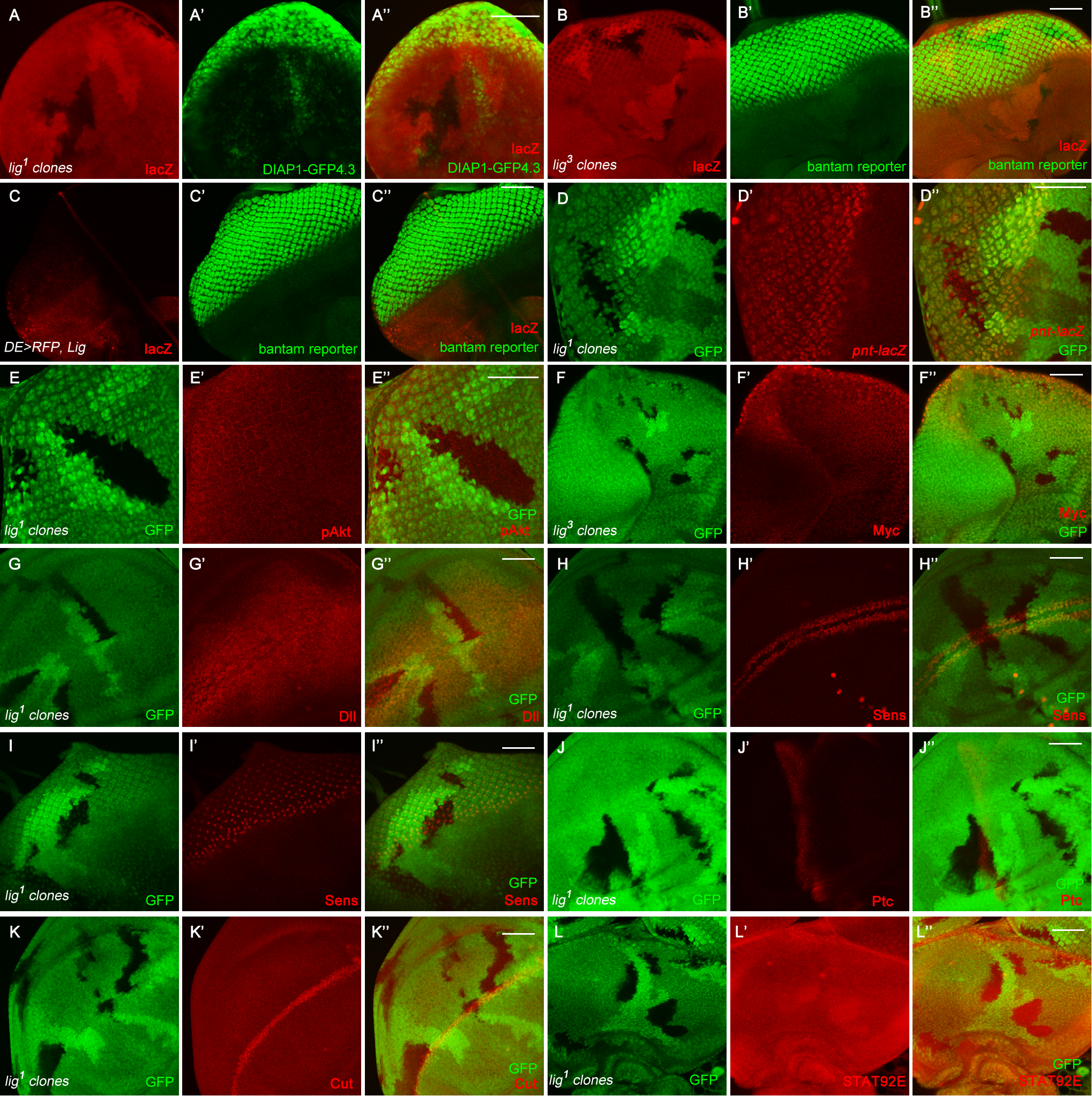

Supplement: Figure S8 — Lig does not regulate bantam miRNA, EGFR signaling, Myc, Hippo signaling, Insulin signaling, Wnt signaling and Hedgehog signaling. (A–A'') lig1 mutant clones (induced with the FLP/FRT system, 72 h old, marked by the lack of lacZ staining in red) in eye imaginal discs of third instar larvae (A and A'') do not display an upregulation of a minimal Hippo response element (DIAP1-GFP4.3; green; A' and A''). Scale bar represents 50 µm. (B–C'') Negatively marked 72 h old lig3 mutant clones (induced with the FLP/FRT system; no lacZ (red); B and B'') and lig overexpressing cells (induced with the Gal4/UAS system using DE-Gal4) marked with RFP (C and C'') in eye imaginal discs of third instar larvae do not impact on a bantam miRNA reporter (B', B'', C' and C''). Scale bar represents 50 µm. (D–K'') Negatively marked 72 h old lig1 mutant clones (induced with the FLP/FRT system; no GFP (green)) in wing (G, G'', H, H'', J, J'', K and K'') or eye (D, D'', E, E'', F, F'', I and I'') imaginal discs do not change expression or localization of pnt-lacZ (D' and D''), pAkt (E' and E''), Myc (F' and F''), Dll (G' and G''), Sens (H', H'', I' and I''), Ptc (J' and J'') and Cut (K' and K''). Scale bars represent 50 µm. (L–L'') Negatively marked 72 h old lig1 mutant clones (induced with the FLP/FRT system; no GFP (green)) in eye imaginal discs stained for STAT92E (L' and L''). Scale bars represent 50 µm. Genotypes: (A) y w hsFLP/y w; FRT42 arm-lacZ/FRT42 lig1; DIAP1-GFP4.3/+ (B) y w hsFLP/y w; FRT42 arm-lacZ/FRT42 lig3; MIR33 bantam reporter/+ (C) y w/y w; UAS-lig/+; MIR33 bantam reporter/DE-Gal4, UAS-RFP (D) y w hsFLP/y w; FRT42 ubiGFP/FRT42 lig1; pnt-lacZ/+ (E, F, G, H, I, J, K, L) y w hsFLP/y w; FRT42 ubiGFP/FRT42 lig1. (TIF) [file pgen.1003598.s008.tif]
